# Mas receptor activation facilitates innate hematoma resolution and neurological recovery after hemorrhagic stroke in mice

**DOI:** 10.1186/s12974-024-03105-8

**Published:** 2024-04-24

**Authors:** Xiangyang Deng, Junwei Ren, Kezhu Chen, Jin Zhang, Quan Zhang, Jun Zeng, Tianwen Li, Qisheng Tang, Jian Lin, Jianhong Zhu

**Affiliations:** 1 Department of Neurosurgery, Wenzhou Municipal Key Laboratory of Neurodevelopmental Pathology and Physiology, The Second Affiliated Hospital and Yuying Children’s Hospital of Wenzhou Medical University, 109, Xueyuan Road, Wenzhou, 325027 Zhejiang China; 2 Department of Neurosurgery, Huashan Hospital, National Center for Neurological Disorders, National Key Lab. for Medical Neurobiology, Institutes of Brain Science, Shanghai Key Lab. of Brain Function and Regeneration, Institute of Neurosurgery, MOE Frontiers Center for Brain Science, Shanghai Medical College, Fudan University, 12 Wulumuqi Zhong Rd, Shanghai, 200040 China; 3 Department of Neurosurgery, The First Affiliated Hospital of Zhengzhou University, Zhengzhou, Henan China; 4 The First Affiliated Hospital of the Naval Medical University, Shanghai, China

**Keywords:** Intracerebral hemorrhage, Mas, AVE0991, Hematoma absorption, Neuroprotection

## Abstract

**Background:**

Intracerebral hemorrhage (ICH) is a devastating neurological disease causing severe sensorimotor dysfunction and cognitive decline, yet there is no effective treatment strategy to alleviate outcomes of these patients. The Mas axis-mediated neuroprotection is involved in the pathology of various neurological diseases, however, the role of the Mas receptor in the setting of ICH remains to be elucidated.

**Methods:**

C57BL/6 mice were used to establish the ICH model by injection of collagenase into mice striatum. The Mas receptor agonist AVE0991 was administered intranasally (0.9 mg/kg) after ICH. Using a combination of behavioral tests, Western blots, immunofluorescence staining, hematoma volume, brain edema, quantitative-PCR, TUNEL staining, Fluoro-Jade C staining, Nissl staining, and pharmacological methods, we examined the impact of intranasal application of AVE0991 on hematoma absorption and neurological outcomes following ICH and investigated the underlying mechanism.

**Results:**

Mas receptor was found to be significantly expressed in activated microglia/macrophages, and the peak expression of Mas receptor in microglia/macrophages was observed at approximately 3–5 days, followed by a subsequent decline. Activation of Mas by AVE0991 post-treatment promoted hematoma absorption, reduced brain edema, and improved both short- and long-term neurological functions in ICH mice. Moreover, AVE0991 treatment effectively attenuated neuronal apoptosis, inhibited neutrophil infiltration, and reduced the release of inflammatory cytokines in perihematomal areas after ICH. Mechanistically, AVE0991 post-treatment significantly promoted the transformation of microglia/macrophages towards an anti-inflammatory, phagocytic, and reparative phenotype, and this functional phenotypic transition of microglia/macrophages by Mas activation was abolished by both Mas inhibitor A779 and Nrf2 inhibitor ML385. Furthermore, hematoma clearance and neuroprotective effects of AVE0991 treatment were reversed after microglia depletion in ICH.

**Conclusions:**

Mas activation can promote hematoma absorption, ameliorate neurological deficits, alleviate neuron apoptosis, reduced neuroinflammation, and regulate the function and phenotype of microglia/macrophages via Akt/Nrf2 signaling pathway after ICH. Thus, intranasal application of Mas agonist ACE0991 may provide promising strategy for clinical treatment of ICH patients.

**Supplementary Information:**

The online version contains supplementary material available at 10.1186/s12974-024-03105-8.

## Introduction

Intracerebral hemorrhage (ICH) is a subtype of stroke with rapid onset, fast progression, high mortality and disability rates, which imposes a huge burden on patients and society [1–3]. While ICH accounts for approximately 10–15% of all reported stroke worldwide, it results in a disability burden that exceeds 50%. Initial hematoma size and progression are important prognostic factors in the primary injury following ICH. Despite clinical progress in surgical procedures for minimally invasive removal of hematoma, several large randomized controlled trials had failed to prove that surgical intervention could significantly improve ICH patient outcome [4–7]. And mounting evidences have demonstrated that ICH leaded to secondary brain injury caused by factors such as neuroinflammation, oxidative stress, and cell apoptosis, which significantly contributed to poor prognosis on these patients [2, 8–10]. Therefore, there is an urgent need to identify the pathogenesis of ICH injury, and to develop effective treatment strategies for these patients. Regulating the function and phenotype of microglia is one of the potential directions.

Microglia are innate immune cells in the central nervous system (CNS). ICH-induced brain injury triggers the activation of microglia in the surrounding areas of injury and infiltration of peripheral macrophages, which then play a role in regulating neuroinflammation, hematoma phagocytosis, and tissue repair processes [11–13]. Previous studies have demonstrated that microglia/macrophages are highly dynamic and capable of exhibiting either pro-inflammatory or regulatory phenotypes during various stages of brain injury [12, 13]. Therefore, it is crucial to identify targets that stimulate the transformation of microglia/macrophages towards an anti-inflammatory, phagocytic, and reparative phenotype. Additionally, elucidating the molecular mechanisms that govern the functional phenotype of these immune cells following ICH is essential. These endeavors have the potential to provide an innovative direction and strategy for the treatment of cerebral hemorrhage.

Although the renin-angiotensin system (RAS) is commonly recognized as a circulating hormone system that controls blood pressure and electrolyte balance, recent researches have revealed the extensive regulatory function of this systemic and tissue-specific response network in various tissues and organs [14, 15]. In the classical RAS system, angiotensin converting enzyme (ACE) promotes the production of Angiotensin II (Ang II), leading to vasoconstriction, inflammation, and cell apoptosis through the AT1 receptor. However, the newly discovered ACE2/Ang-(1–7)/Mas axis within the RAS system is crucial for maintaining nervous system homeostasis by mediating vasodilation, anti-inflammatory effects, and anti-apoptotic actions. This pathway effectively counteracts the effects of the ACE/Ang-II/AT1 axis [15–17]. Thus, targeting the local brain RAS system is a promising approach to treating CNS injury.

As an important branch of the local RAS system in the brain, ACE2 can stimulate downstream Mas receptors by converting Ang II into Ang-(1–7), which is involved in antioxidant stress and anti-inflammatory processes [15, 18]. Previous researches have also shown that the Ang-(1–7)/Mas axis could play a neuroprotective role in various CNS diseases [19–23]. However, the role of the Mas receptor in the setting of ICH remains to be elucidated. Some studies have indicated that the activation of Mas receptors could stimulate the production of arachidonic acid and nitric oxide, as well as the activation of phospholipase A2 (PLA2), phosphatidylinositol 3-kinase/protein kinase B (PI3K/Akt), mitogen-activated protein kinase (MAPK), and protein kinase (PKA) [15, 21]. As a proto-oncogene, Akt plays a crucial role in various biological processes. Disruptions to the Akt signaling pathway are associated with multiple human diseases, such as cancer, cardiovascular disease, and neurological disorders [24, 25]. And it’s reported that phosphorylated Akt could upregulate the expression of nuclear factor E2-related factor 2 (Nrf2), which was involved in antioxidant and cytoprotective functions, inhibition of inflammation, and modulation of microglial functional phenotype [26–29]. Given the close relationship between the aforementioned proteins and Nrf2’s pivotal regulatory function, it is plausible that Mas receptor activation could mediate hematoma clearance and neuroprotection partially via the Akt/Nrf2 pathway in ICH.

In the present study, we reported that Mas activation promoted hematoma resolution, attenuated neuroinflammation and alleviated neurological dysfunction through regulation of microglial functional phenotype via the Akt/Nrf2 signaling pathway following ICH.

## Methods

### Animals

Male C57BL/6 mice, aged 8–10 weeks and weighing between 25-30 g, were purchased from Zhejiang Vital River Laboratory Animal Co., Ltd. The mice were kept in a controlled environment, with a regulated temperature and humidity, and a 12-h light/dark cycle. All animal experiments described in this study were carried out in compliance with the Animal Care and Use Committee regulations.

### Experimental design

I experimental design was depicted in Additional file 1: Part S1.

### ICH model

ICH model was established by injection of collagenase into mice striatum [30]. Briefly, a 0.3% solution of pentobarbital sodium was utilized for anesthesia. Prior to injection, the mice were weighed and then received an intraperitoneal injection of 0.1–0.2 ml/10 g of the anesthetic agent. After complete anesthesia, mice were fixed onto the brain stereotaxic frame, and the head retainer was adjusted to maintain the horizontal position of the mouse’s head. The scalp hair was shaved, and the surgical area was cleaned with an alcohol swab. A 0.5–1 cm longitudinal incision was made to expose the skull, and the anterior fontanelle was uncovered. The needle of a Hamilton syringe was then inserted through a burr hole into the right basal ganglia at coordinates: 0.5 mm anterior, 2.2 mm lateral, and 3.5 mm ventral to the bregma. After confirming the injection site, the skull was opened with a grinding drill, and 0.5 μl of normal saline solution containing 0.0375U collagenase was injected into the right striatum of the mice at a uniform rate of 0.1 μl/min using a microinjection pump. Once the injection was completed, the microsyringe remained in situ for 5 min before being slowly removed. The burr hole was sealed with bone wax, and skin incision was then closed. The operation procedure was identical in the sham operation group, except no collagenase was administered. The operation was performed under sterile conditions, and the mice’s body temperature was maintained at 37 ℃ using a heating pad. Following the operation, mice were resuscitated with 0.4 ml sterile saline, and their body temperature was sustained. Mice were monitored closely until fully recovered from anesthesia. Once resuscitated, mice had free access to food and water.

### Drug administration

According to the previous research literature, intranasal administration can improve the central utilization rate and reduce the peripheral side effects by delivering the drug to the brain via the olfactory nerve and ethmoid sinus, and it can be administered repeatedly for a long time [31]. In order to facilitate clinical transformation, this study was conducted by intranasal administration. AVE0991 at dosages of 0.3, 0.9, and 2.7 mg/kg dissolved in 10% dimethyl sulfoxide (DMSO) mixed with corn oil and intranasally administered 1 h after ICH for 3 consecutive days as previously reported [23]. A779 (3 nmol in 2 µL), a selective inhibitor of Mas receptor, was dissolved in PBS and administered intracerebroventricularly (i.c.v.) 1 h before ICH injury. Intracerebroventricular administration was performed as previously described [32]. Intracerebroventricular injection was executed at a flow rate of 0.25 μL/min with the aid of the following coordinates referenced from bregma: 0.3 mm posterior, 1 mm right lateral, and 2.3 mm ventral. ML385 (30 mg/kg), a selective inhibitor of Nrf2, was dissolved in 5%DMSO and administered intraperitoneally at 1 h prior to ICH induction.

### Neurobehavioral tests

In this study, the modified neurological deficit score (mNSS), corner turning test, and forelimb placement test (FP test) were utilized to assess the short-term neurofunctional behavior of mice (1, 3, 5, and 7 days after ICH) [32, 33]. The medium- and long-term neurofunctional behavior of mice was evaluated through the open field test (14 days after ICH) and the Morris water maze test (23–28 days after ICH).

The overall score of the mNSS was 18, which was determined by assessing movement, sensation, balance, and reflexes in mice. The severity of symptoms was directly proportional to the score obtained. In the corner turning test, mice were observed over 10 trials and a score was determined by calculating the percentage of right turns out of 10 trials. For the forelimb placement test, the positioning of the left forelimb on the countertop was recorded upon stimulation of the vibrissae. The percentage of times the left forelimb was positioned correctly out of ten consecutive vibrissae stimulations was then calculated.

For the open field test, experimental mice were randomly placed at the center of the test box and recorded video for 10 min. After each experiment, the total distance moved and central area distance moved by the mice were recorded and calculated within the 10-min timeframe.

The Morris water maze test was performed on days 23–28 after ICH to evaluate the spatial learning and memory capabilities, which has been previously reported [32, 34]. The water maze device consisted of a circular cylindrical pool that was divided into four quadrants and equipped with an image acquisition system. The water in the pool was colored milky white, and a 10 cm platform with a diameter ranging from 0.5 to 1 cm was situated in one of the quadrants, with azimuth marks positioned around the pool to serve as cues for the mice. The experimental mice were allowed to swim for up to 60 s, starting at different quadrants in each trial and stayed on the platform for 15 s. On day 28, the platform was removed, and the mice were placed into the pool from the opposite side of the original platform quadrant. The complete movement tracks of the mice within 60 s were recorded, and the percentage of stay time and the number of platform crossings in the quadrant of the original platform were subjected to statistical analysis.

### Liquid Chromatograph-Mass Spectrometer (LC–MS) analysis

The brain samples were prepared as previously described [23]. For LC–MS analysis, the entire brain was weighed and mixed with 1.3 mL of methanol. After grinding the tissue for 5 min and gently scrolling for 5 min more, the sample was transferred to a 1.5 mL centrifuge tube and subjected to 10 min of centrifugation at 13,000 rpm. The resulting supernatant was carefully collected and filtered using a 0.22 μm filter membrane before being injected into the LC–MS system. The mass spectrometer (ThermoFisher) was set to the positive ion scanning mode and selective reaction monitoring (SRM), while the chromatograph (ThermoFisher) adopted the Welch Ultimate XB-C8 150 4.6 mm, 5 μm column. The chromatogram was collected and analyzed using Xcalibur software (ThermoFisher).

### Hematoma volume and hemoglobin content measurement

The measurement of hematoma volume was conducted in accordance with established protocols as previously reported [32]. This assessment was performed at intervals of 3 and 7 days after inducing ICH. In brief, the mice were placed under deep anesthesia and transcardially perfused with 4 °C phosphate-buffered saline (PBS). The entire brain was removed and sliced coronally at 1-mm intervals, with each section photographed. The hematoma volume was calculated using the formula: hematoma volume (mm^3^) = area of each section (mm^2^) × number of sections × thickness (mm), as determined using the Image J software.

Hemoglobin assay was carried out using Drabkin's reagent to quantify hematoma according to previously published methods [32]. Following homogenization and ultrasonic lysis of the brain sections, the samples were centrifuged to obtain the supernatant. 0.4 mL of Drabkin’s reagent (Sigma-Aldrich) was added to 0.1 mL of supernatant and allowed to react for 15 min at room temperature. The hemoglobin absorbance was measured by a spectrophotometer (ThermoFisher) at 540 nm. The data was then calculated as a ratio to the sham group, with the preset value of 1 assigned to the sham group [35].

### Brain water content measurement

After inducing deep anesthesia, the mice were quickly decapitated, and the entire brain tissues were immediately taken out. The olfactory bulb was removed. The brain tissue was then divided into left and right hemispheres as well as the cerebellum, which were weighed using an electronic balance and recorded as wet weight. Subsequently, the three portions were placed in a constant temperature oven at 100 ℃ for 3 days before being weighed again and recorded as dry weight. The brain water content was calculated as (wet weight-dry weight)/wet weight*100%.

### Enzyme‑linked Immunosorbent Assay (ELISA)

Serum and brain tissue levels of Ang-(1–7) were quantified using a commercially available Mouse Angiotensin 1–7 ELISA kit (CUSABIO) in accordance with the manufacturer's instructions. Each ELISA analysis was performed in triplicate. To quantify Ang-(1–7) levels, the absorbance of samples was read and analyzed at 450 nm using a spectrophotometric plate reader (ThermoFisher).

### Quantitative real-time polymerase chain reaction (q-PCR)

The surrounding brain tissues of the hematoma were used for q-PCR analysis. Total RNA was extracted using the RNA extraction reagent (TRIzol, Invitrogen) following the manufacturer's instructions. Subsequently, cDNA synthesis and subsequent qPCR were performed using the Servicebio® RT First Strand cDNA Synthesis Kit (Servicebio) and SYBR Green qPCR Master Mix (without ROX, Servicebio). The synthetic primer sequences are listed in Additional file 1: Part S2.

### Western Blot analysis

Under deep anesthesia, mice were transcardially perfused with cold PBS, and brains were extracted and surrounding tissues of the hematoma were dissected. Total proteins of brain tissues were extracted using SD-001/SN-002 Minute™ Total Protein Extraction Kit (Invent Biotechnologies). Equal amounts of protein were loaded on an SDS-PAGE gel and run using electrophoresis and then transferred to a nitrocellulose membrane. The membrane was blocked with 5% nonfat blocking grade milk and then incubated overnight at 4 °C with the following primary antibodies: rabbit anti-MPO (1:1000, ab208670, Abcam), rabbit anti-IL-1β (1:1000, 12,426, Cell Signaling Technology), rabbit anti-Bax (1:5000, 50,599–2-Ig, Proteintech), rabbit anti-Bcl-2 (1:2000, 26,593–1-AP, Proteintech), rabbit anti-p-Akt (Ser473, 1:1000, #4060, Cell Signaling Technology), rabbit anti-Akt (1:10,000, 60,203–2-Ig, Proteintech), rabbit anti-Nrf2 (1:1000, 12,721, Cell Signaling Technology, USA), rabbit anti-iNOS (1:1000, ab178945, Abcam), rabbit anti-CD163 (1:1000, 16,646–1-AP, Proteintech). The membranes were incubated with a rabbit monoclonal antibody anti-β-actin (1:20,000, AC026, ABclonal) as an internal reference. The appropriate secondary antibodies (1:5000, Cell Signaling Technology) were chosen for 2 h of incubation at room temperature. The density of the bands was measured by ImageJ software (NIH) and expressed as a ratio between the intensity of the target band and that of β-actin band.

### Immunofuorescence staining

After being anesthetized deeply, mice were transcardially perfused with cold PBS and 4% paraformaldehyde. Then the brains were removed and placed successively in 4% paraformaldehyde, 20% sucrose, and 30% sucrose for complete fixation and dehydration. The embedded brain tissues were sliced along the coronal plane with a thickness of 30 μm using a constant temperature frozen slicer. After being washed in PBS and PBS + 0.3% Triton, coronal sections were incubated in PBS + 1% Triton to break the cell membrane and blocked with immunofluorescence blocking solution for 1 h. The brain sections were then incubated overnight at 4 °C with primary antibodies including: rabbit anti-Mas (1:100, NBP1-78,444, NOVUS), rat anti-iba1 (1:500, ab283346, Abcam), rabbit anti-NeuN (1:400, ab279297, Abcam), rabbit anti-GFAP (1:400, ab279291, Abcam), rabbit anti-MPO (1:100, ab208670, Abcam), rabbit anti-IL-1β (1:200, 12,426, Cell Signaling Technology), rabbit anti-CD16/32 (1:500, ab223200, Abcam), rabbit anti-iNOS (1:200, 18,985–1-AP, Proteintech), rabbit anti-Arg1 (1:200, 16,001–1-AP, Proteintech), rabbit anti-CD163 (1:200, 16,646–1-AP, Proteintech). Sections were then washed with PBS three times with 10 min intervals, and incubated with the corresponding secondary antibody for 1 h at room temperature. Nuclear staining was conducted by 4′,6-diamidino-2-phenylindole (DAPI). Sections were then visualized, and photographed under a Nikon microscope.

### TUNEL staining

For quantification of neuronal apoptosis, double staining of neuron marker NeuN and TUNEL staining was conducted using One Step TUNEL Apoptosis Assay Kit (Beyotime) according to the manufacturer’s instructions at 3d after ICH. The number of TUNEL-positive neurons was counted manually in the peri-hematoma area. Data were expressed as the ratio of TUNEL-positive neurons (%).

### FJC staining

At 3 days post ICH, degenerating neurons were assessed using a modified FJC Ready-to-Dilute Staining (Millipore) with FJC staining, as previously described [36]. According to the manufacturer’s instructions, the brain slices were baked in a 37 ℃ oven for 2–3 days, and consecutively incubated in solutions A, B, and C, then thoroughly washed. The slices were then baked at 50 ℃ for 10 min and incubated in xylene for 2 min. Once dried, the slices were sealed with neutral resin before being analyzed and photographed under a Nikon confocal microscope after a 5-min interval. The FJC-positive cells were counted in the peri-hematoma regions of the brain. The data were averaged and expressed as positive cells/mm^2^.

### Nissl staining

In this study, the Nissl staining reagent (Servicebio) was utilized to evaluate neuronal degeneration in the hippocampus following ICH. Brain slices were washed in a container filled with PBS, and subsequently incubated with Nissl’s dye solution for 3 min. Then, the brain slices were rinsed with water until it ran clear and dried in an oven at 65 ℃. After being completely dried, the slices were carefully mounted with neutral resin and photographed with a microscope. Neuronal degeneration in the hippocampus cornu ammonis 1(CA1) region was observed and quantified.

### Cell culture and vitro ICH model

Microglial BV2 cells and neuronal HT22 cells were cultured in a complete medium containing DMEM with high glucose, 10% FBS and 1% penicillin/streptomycin at 37 °C under 5% CO2. Cells treated with a complete medium containing 10 μM hemin (Sigma-Aldrich, Germany) and vehicle for 24 h to mimic the ICH model in vitro. AVE0991-treated conditioned medium of BV2 cells were used to culture HT22 cells to elucidate whether AVE0991 influences neuronal apoptosis through microglial cells. Co-treatment with 10 μM AVE0991 was used to evaluate the protective effects of Mas activation.

### Dead/live assay

The Calcein/PI Cell Viability/Cytotoxicity Assay Kit (Beyotime) was used to conduct dead/live experiment according to the instruction. HT22 cells were washed with PBS and then incubated with Calcein/PI working solution at 37 °C for 30 min darkness. Cells were washed again and observed under a fluorescence microscope.

### Statistical analysis

All the data in this study were reported as the mean and standard deviation (SD). Results were analyzed by Student’s t-test, One-way ANOVA with Tukey’s post hoc test or Two-way ANOVA with Tukey's post hoc test where appropriate. A P-value < 0.05 was considered statistically significant.

## Results

### Animal mortality

The mortality rate in this study was 3.55% (12/338). A total of 12 mice died in ICH group and there was no morality in sham groups.

### Time course and cellular expression of mas receptor after ICH

**Fig. 1:**
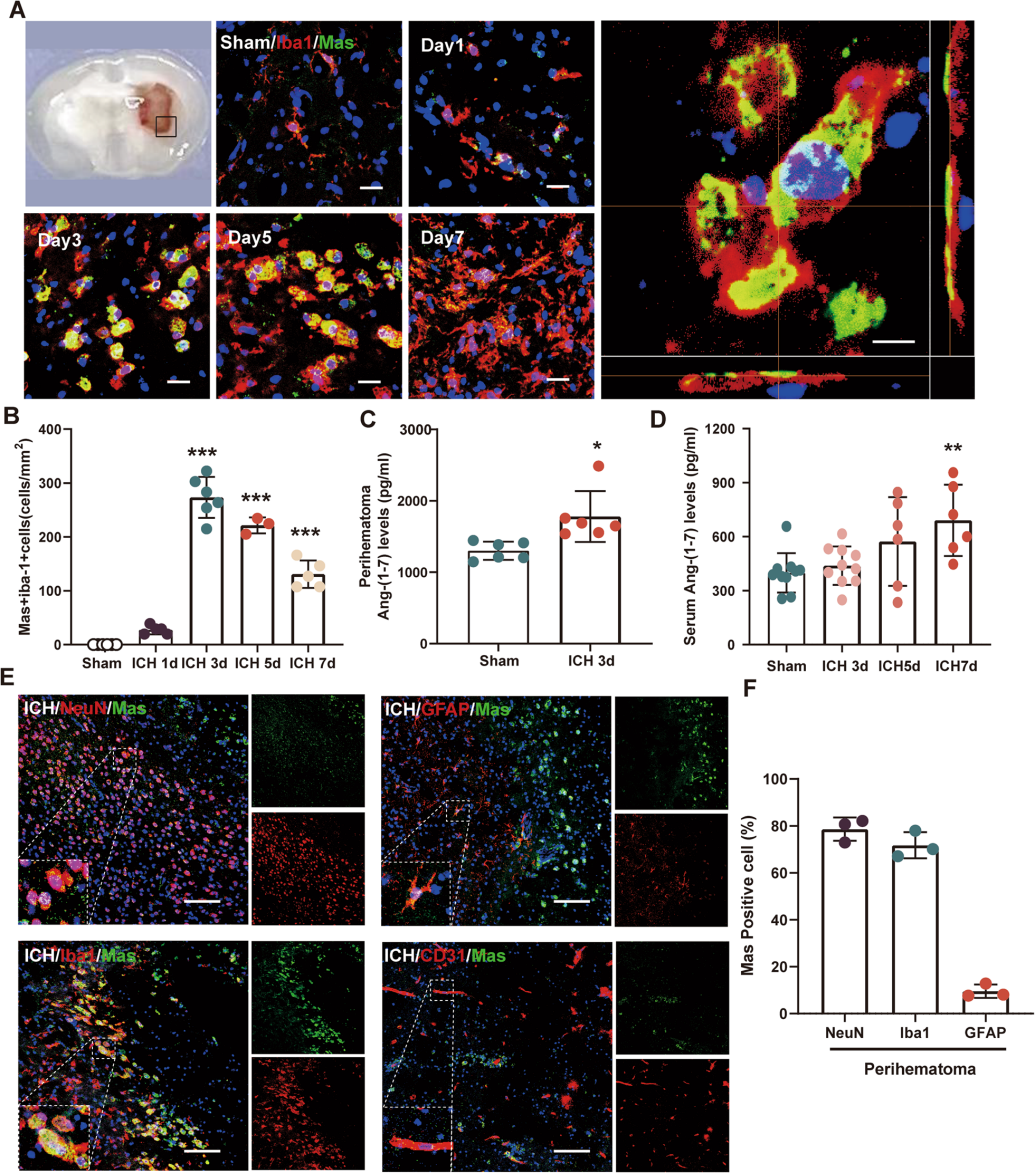
Expression profile of Mas and Ang-(1–7) after ICH. **A** Immunofluorescence assessment of Mas (green) in microglia/macrophages (iba1 + , red) in the sham group and the perihematomal area of ICH group. Nuclei were stained with DAPI (blue). Scale bar1 = 20 μm, scale bar2 = 10 μm. **B** Quantitative analyses of Iba1 + /Mas + microglia/macrophage and in the perihematomal area after ICH, ***P < 0.001 vs. ICH 1d, n = 3–6/group. **C** Comparison of perihematomal Ang-(1–7) levels between Sham and ICH mice at 3 days after ICH surgery. *P < 0.05 vs. Sham, n = 6/group. **D** Comparison of serum Ang-(1–7) levels between Sham and ICH mice at 3, 5, and 7 days after ICH surgery. **P < 0.01 vs. Sham; n = 10 in Sham and ICH-3d groups, n = 6 in ICH-5d and ICH-7d groups. **E** Representative images of colocalization of Mas with microglia (iba1, red) astrocytes (GFAP, red), neurons (NeuN, red) and vascular endothelial cell (CD31, red) in the perihematomal area of ICH (3 day) group. Nuclei were stained with DAPI (blue). Scale bar = 100 μm, n = 3/group. **F** The proportional expression of Mas in neurons, microglia and astrocytes surrounding the hematoma

Although alterations in Mas expression have been reported in cases of subarachnoid hemorrhage, it is currently unclear whether the Mas axis is affected in instances of ICH. Given that the primary cells responsible for hematoma clearance are microglia/macrophages, we performed to assess Ang-(1–7)/Mas expression and found that Mas was expressed considerably in activated microglia/macrophages via double immunofluorescence staining of Iba1 and Mas. By examining different time points, it was noted that the peak expression of Mas receptor in microglia/macrophages was observed at approximately 3–5 days, followed by a subsequent decline (Fig. 1A and B). ELISA was performed to evaluate the concentration of Ang-(1–7) in the brain tissue around hematoma in the Sham group and the 3-day ICH group, and our findings indicated a significant increase in Ang-(1–7) levels at 3 days after ICH (Fig. 1C). Early identification of serum biomarkers for poor prognosis is essential for optimizing clinical diagnosis and treatment. In this regard, we investigated whether Ang-(1–7) could serve as a prognostic marker by detecting its serum levels following ICH in mice. Our ELISA results indicated that there was no significant difference in Ang-(1–7) expression between the Sham group and the 3-day ICH group (P > 0.05, Fig. 1D); however, further multitime point determination revealed a progressively elevated Ang-(1–7) levels, manifesting as a significant ascent on the 7 day after ICH (P < 0.05, Fig. 1D).

To further investigate the distribution characteristics of Mas receptor in the perihematomal region, double immunofuorescence staining revealed that the expression of Mas was predominantly observed in microglia and neurons with little expressions noted in astrocytes surrounding the hematoma (Fig. 1E and F). Additionally, we also observed a minimal expression of Mas in the microvessels (Fig. 1E and F).

### LC–MS analysis proved that intranasal administration of AVE0991 could effectively enter the brain

**Fig. 2:**
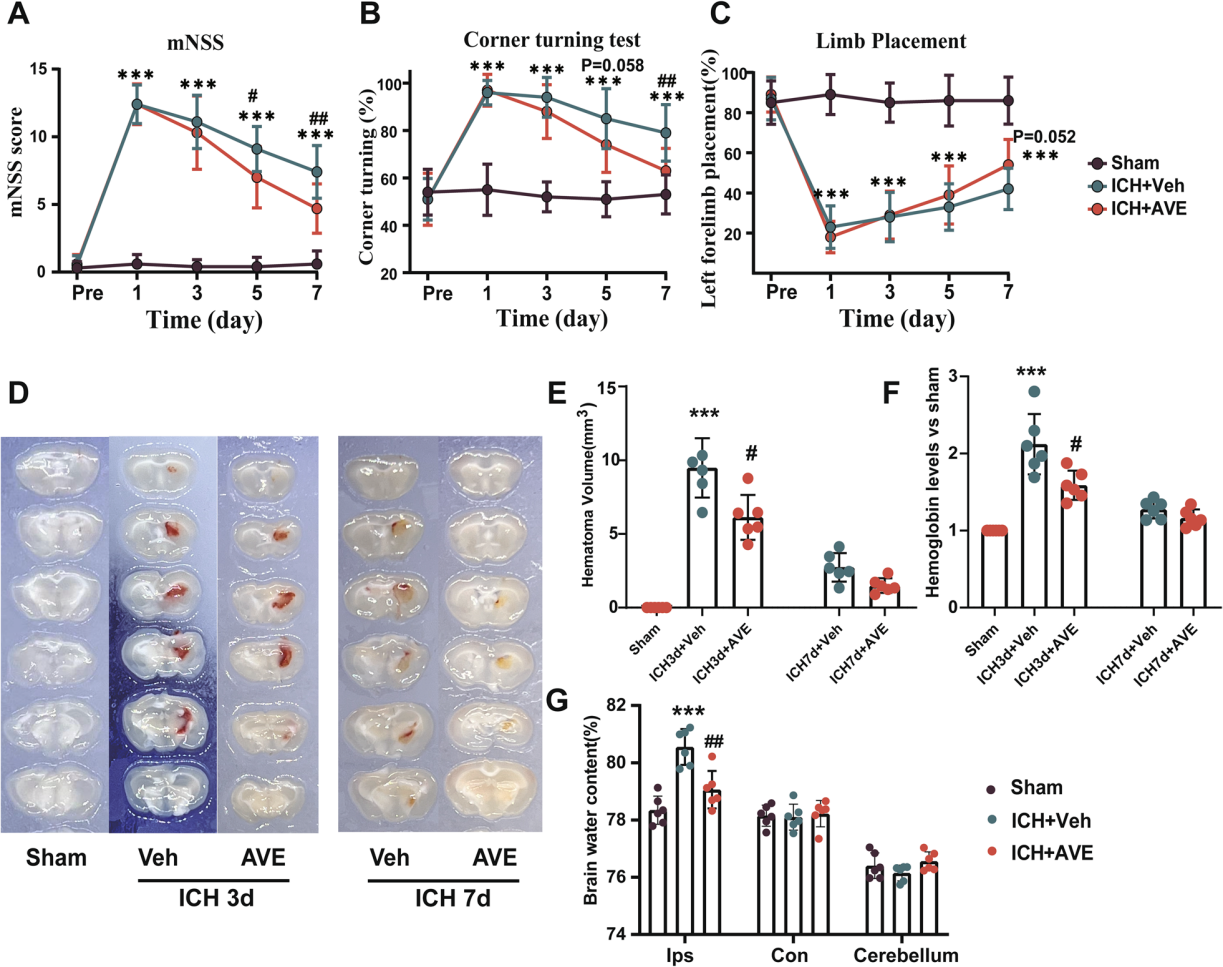
The effects of AVE0991 on hematoma absorption and short-term neurobehavior outcomes after ICH. **A–C** Modified neurological deficit score (mNSS), corner turning tests, and forelimb placement tests at 1, 3, 5 and 7 days after ICH. **D** Representative photograph of brain sections. **E**–**F** quantitative analyses of hematoma volume and hemoglobin level, **G** brain water content at 3 days after ICH. Data was represented as mean ± SD. ***P < 0.001 vs Sham; ^#^P < 0.05, ^##^P < 0.01, ^###^P < 0.001 vs. ICH + Vehicle. n = 6/group. Veh, Vehicle; AVE, AVE0991

LC–MS analysis was conducted to confirm whether AVE0991 entered the brain tissue following intranasal administration (Additional file 1: Fig. S2). Gradient elution via liquid chromatography (LC) was used to detect the samples (Additional file 1: Fig. S2A–D). Both the standard sample and the examined brain tissue exhibited peaks at 3.95 min (Additional file 1: Fig. S2 A and C). Further analysis via mass spectrometry (MS) revealed similar fragment peaks in both the standard sample (Additional file 1: Fig. S2B) and the tested brain tissue (Fig. 2S D), specifically two fragment peaks of 536.20 m/z and 290.95 m/z (Additional file 1: Fig. S2E and F). These fragment peaks were identified as the decomposition products of AVE0991, indicating that AVE0991 effectively entered the brain tissue following intranasal administration.

### AVE0991 treatment attenuated short-term neurobehavioral deficits, promoted hematoma resolution, and reduced brain edema after ICH

We evaluated the efficacy of three different doses of AVE0991 (0.3 mg/kg, 0.9 mg/kg, 2.7 mg/kg) to determine the optimal dose. In neurobehavioral tests, AVE0991 treatment at both middle dose (0.9 mg/kg) and high dose (2.7 mg/kg) significantly improved neurological performance (mNSS score) at 7 days after ICH (Additional file 1: Fig. S3). And we also observed that the high-dose group did not yield any additional neurological functional improvement benefits compared to the middle-dose group. Based on these results, AVE0991 0.9 mg/kg dose was used for the rest of the experiments in this study.

AVE0991 0.9 mg/kg dose were then utilized to evaluate the extent of short-term neurological impairment following ICH via various methods of neurological function assessment. And we found that mice treated with AVE0991 exhibited significantly better neurological function compared to those in the ICH + Vehicle group. Specifically, while both the mNSS score and the corner test score showed a decrease at the 5 and 7 days after ICH (P < 0.05, Fig. 2A and B), while the score for the forelimb placement test demonstrated an upward trend (7 days: P = 0.052, Fig. 2C).

To investigate the impact of Mas receptor activation on hematoma clearance, mice were sacrificed at 3 and 7 days after ICH to measure hematoma volume and brain hemoglobin content. Our results demonstrated that AVE0991 treatment significantly reduced the hematoma volume at both the 3 and 7 days after ICH (P < 0.05, Fig. 2D, E). The hemoglobin content results at 3 days after ICH were consistent with the reduction in hematoma volume (P < 0.05, Fig. 2F). However, no significant difference was observed between the two groups at the 7 days after ICH (P > 0.05, Fig. 2F).

The AVE0991 treatment group also showed prominently lower brain water content in the hemorrhagic hemisphere as compared to the ICH + Vehicle group (P < 0.05, Fig. 2G), while there was no significant difference in the brain water content of the contralateral hemisphere and cerebellum between the two groups (P > 0.05, Fig. 2G), which indicated the effectiveness of AVE0991 in reliving brain edema.

### AVE0991 treatment improved long-term neurobehavioral outcomes as well as reduced hippocampal CA1 neuronal loss after ICH

**Fig. 3:**
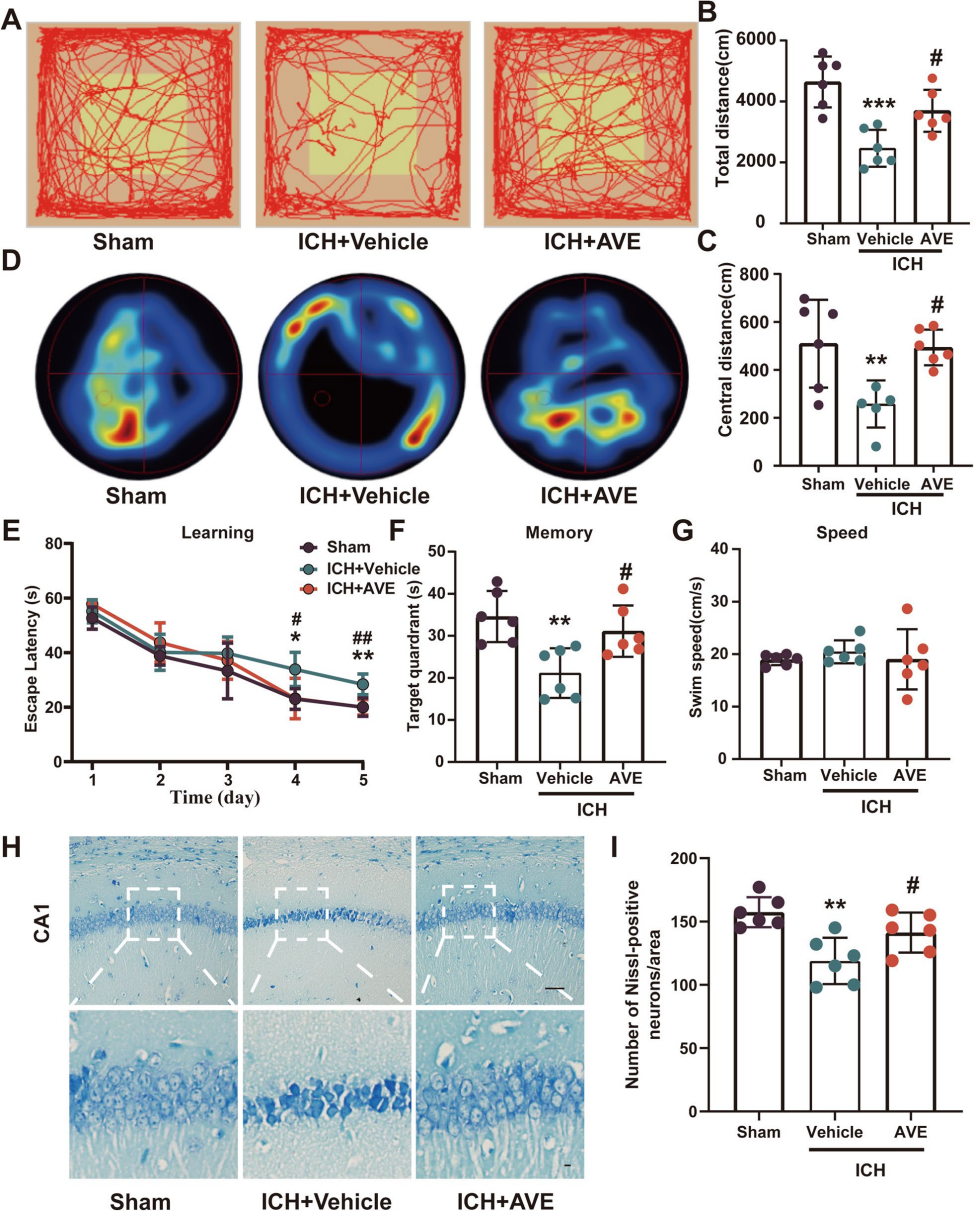
Effects of Mas activation with AVE0991 on long-term outcomes after ICH. **A** The representative mouse open field test during test section. **B**, **C** Total distance and central distance in open field tests after ICH. **D** Representative heatmaps of the probe trial. **D**–**F** Escape latency, target quadrant and swim speed of Morris water maze after ICH. **H** Representative Nissl staining pictures in CA1 hippocampus at 28 days after ICH, scale bar1 = 100 μm, scale bar2 = 10 μm. **I** Quantitative analysis of surviving neurons in the CA1 area of the affected hippocampus at day 28 s after ICH. Data was represented as mean ± SD.**P < 0.01, ***P < 0.001 vs. Sham; ^#^P < 0.05 vs. ICH + Vehicle, n = 6/group. Veh, Vehicle; AVE, AVE0991

The autonomic activity and exploratory behavior of mice at the 14 days after ICH were evaluated using the open field test. The results revealed that mice administered with AVE0991 exhibited significantly improved neurological function compared to those in the ICH + Vehicle group. Specifically, the ICH + AVE0991 group showed an improvement in the total distance of movement (P < 0.05, Fig. 3A and B) and the distance of movement in the central area (P < 0.05, Fig. 3A and C) compared to the ICH + Vehicle group in the open field test.

Morris water maze test was used to test the spatial learning and memory ability of mice after ICH. In the learning test, compared with the Sham group, the mice in the ICH group showed obvious learning ability impairment, which was characterized by a longer time to find the platform (P < 0.05, Fig. 3D and E). After ICH, the AVE0991-treated mice spent less time than the ICH + Vehicle group in finding the hidden platform during training (days 4 and 5, P < 0.05, Fig. 3D and E). In the memory test, the mice in the AVE0991 treatment group stayed longer in the target quadrant than those in the ICH + Vehicle group (P < 0.05, Fig. 3F). And there was no significant difference in swimming speed among the three groups (P > 0.05, Fig. 3G), which indicating that the effect of AVE0991 on cognitive function recovery is not due to the difference in swimming ability or motor performance. Taken together, these data confirmed an impaired spatial learning and memory function in ICH mice, which could be markedly ameliorated via post-intervention treatment with AVE0991.

We further performed Nissl staining on the brain slices of mice 28 days after ICH to evaluate the survival of neurons in the ipsilateral hippocampal CA1 region. The results revealed a lower number of neurons in the ipsilateral hippocampus following ICH compared to the Sham group (P < 0.05, Fig. 3H and I), while the survival of neurons in the hippocampus increased significantly after AVE0991 treatment (P < 0.05, Fig. 3H and I), in line with long-term behavioral outcomes.

### AVE0991 treatment alleviated neuronal apoptosis after ICH

**Fig. 4:**
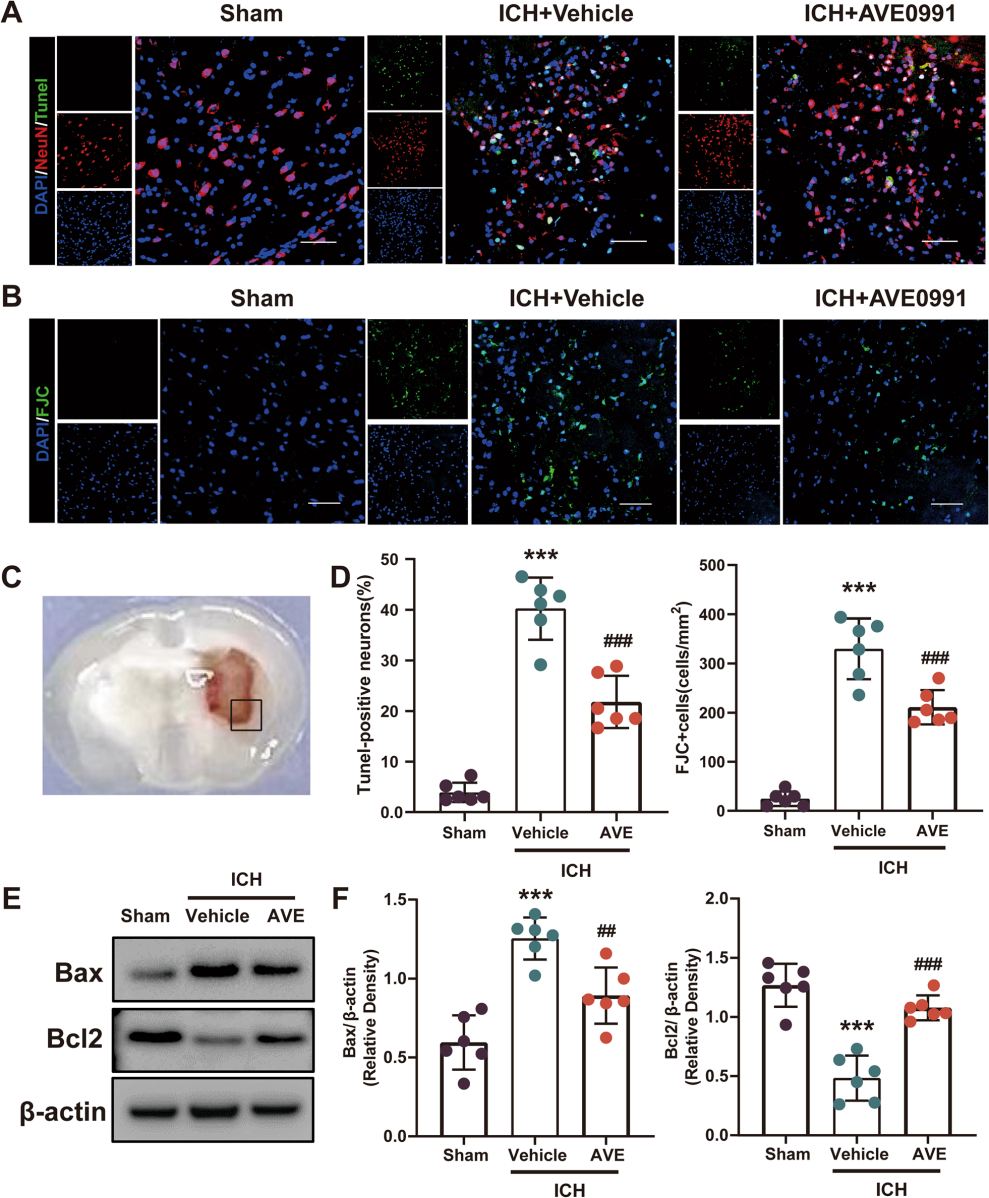
Effects of Mas activation with AVE0991on neuronal apoptosis and neuronal apoptotic molecular markers after ICH. **A**, **B** Representative images of the co-localization of TUNEL (green) with neurons (NeuN, red) and FJC (green) staining in the perihematomal area at 3 days after ICH. Scale bar = 50 μm. **C** Regions of interest areas in the perihematomal region. **D** Quantitative analyses of TUNEL and FJC-positive cells in the perihematomal area at 3 days after ICH (n = 6/group). **E**, **F** Representative western blot bands and quantitative analyses of Bax and Bcl-2 protein levels at 3 days after ICH. Data was represented as mean ± SD. ***P < 0.001 vs. Sham; ^##^P < 0.01, ^###^P < 0.001 vs. ICH + Vehicle, n = 6/group

We have found that the intranasal administration of AVE0991 effectively improved neurological function impairment in ICH mice. To further verify the neuroprotective effect of AVE0991 treatment, TUNEL staining and FJC staining were employed to assess the neuron damage surrounding the hematoma (Fig. 4A–D). The results showed that neuronal apoptosis around hematoma was significantly increased after ICH compared with Sham group, and decreased by about 50% after AVE0991 treatment (P < 0.05, Fig. 4A and D). The results of FJC staining were also similar, that is, AVE0991 treatment could significantly reduce the number of FJC positive cells around the hematoma (P < 0.05, Fig. 4B and D). Further WB analysis showed that the expression of Bax, a neuron pro-apoptotic marker, was significantly decreased after treatment with AVE0991 (P < 0.05, Fig. 4E and F), while the expression of Bcl2, a neuron pro-survival marker, was significantly up-regulated (P < 0.05, Fig. 4E and F), which were consistent with the results of immunofluorescence staining.

### AVE0991 treatment inhibited neutrophil infiltration and inflammatory cytokine release after ICH

**Fig. 5:**
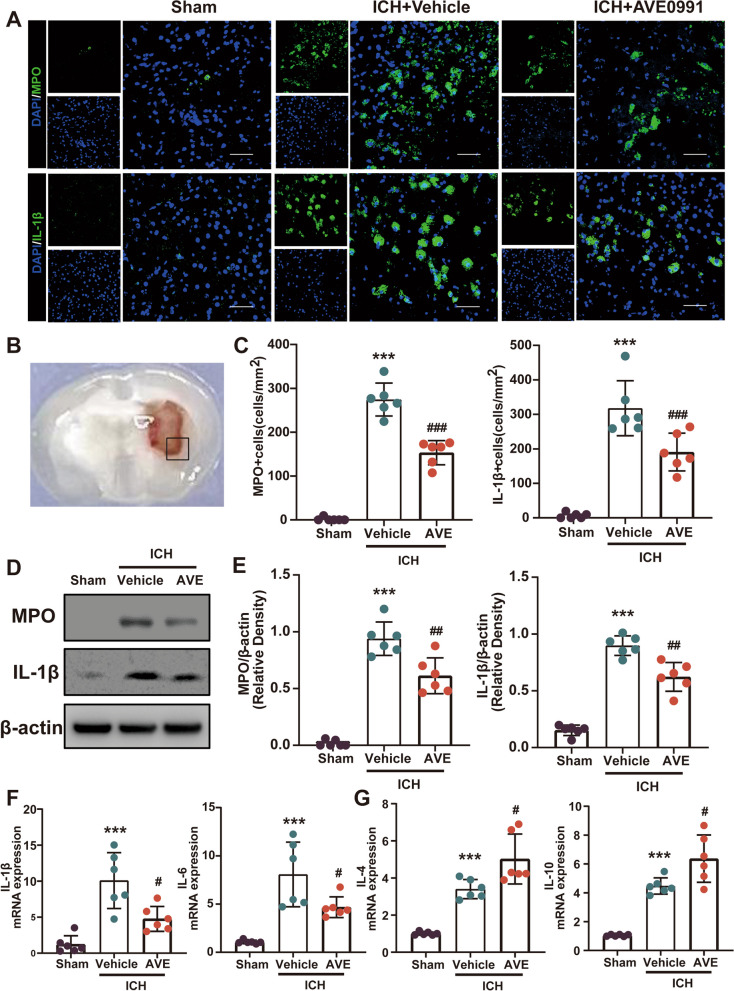
Effects of Mas activation with AVE0991 on neutrophil infiltration and inflammatory cytokine release after ICH. **A** Representative images of immunofuorescence staining of MPO (green) and IL-1β (green) in the perihematomal area at 3 days after ICH. Scale bar = 50 μm. **B** Regions of interest areas in the perihematomal region. **C** Quantitative analyses of MPO and IL-1β-positive cells in the perihematomal area at 3 days after ICH, n = 6/group. **D**, **E** Representative Western blot bands and quantitative analyses of MPO and IL-1β protein levels at 3 days after ICH. **F**, **G** mRNA levels were evaluated including CD16, CD32, IL-1β, iNOS, IL-6, and CD11b. Pro-inflammatory cytokines-associated mRNA levels were evaluated including IL-1β and IL6. Anti-inflammatory cytokines-associated mRNA levels were evaluated including IL-4 and IL10. Data were represented as mean ± SD. ***P < 0.001 vs. Sham group; ^#^P < 0.05, ^##^P < 0.01, ^###^P < 0.001 vs. ICH + Vehicle group. n = 6/group

In this study, peripheral neutrophil infiltration and expression of the pro-inflammatory factor IL-1β were assessed via immunofluorescence staining of MPO and IL-1β in brain slices at 3 days post ICH (Fig. 5A–C). The results revealed a significant increase in MPO and IL-1β positive cells around the hematoma after ICH, indicating a severe inflammatory reaction. After treatment with AVE0991, the number of MPO and IL-1β positive cells in the perihematomal area showed significant reduction (P < 0.05, Fig. 5A and C). In addition, WB analysis showed a significant decrease in MPO and IL-1β expression in the tissue surrounding the hematoma after AVE0991 treatment (P < 0.05, Fig. 5D and E), which aligned with the immunofluorescence results.

To further assess the impact of AVE0991 on inflammatory factor secretion, qPCR was utilized to measure the expression of inflammatory factors in the tissue surrounding the hematoma 3 days after ICH. As depicted in Fig. 5, there was a significant decrease in the expression of IL-1β (P < 0.05, Fig. 5F) and IL-6 (P < 0.05, Fig. 5F) mRNA, both of which were pro-inflammatory cytokines, in the ICH + AVE0991 group compared to the ICH + Vehicle group. Furthermore, the expression of anti-inflammatory-related IL-4 (P < 0.05, Fig. 5G) and IL-10 (P < 0.05, Fig. 5G) mRNA significantly increased in the ICH + AVE0991 group. These findings suggested that AVE0991 treatment could effectively decrease the expression of pro-inflammatory cytokines in ICH mice, normalize their expression, and promote the expression of anti-inflammatory cytokines.

### AVE0991 treatment increased the phenotypic switch of microglia/macrophage from pro‑inflammatory to regulatory phenotype

**Fig. 6:**
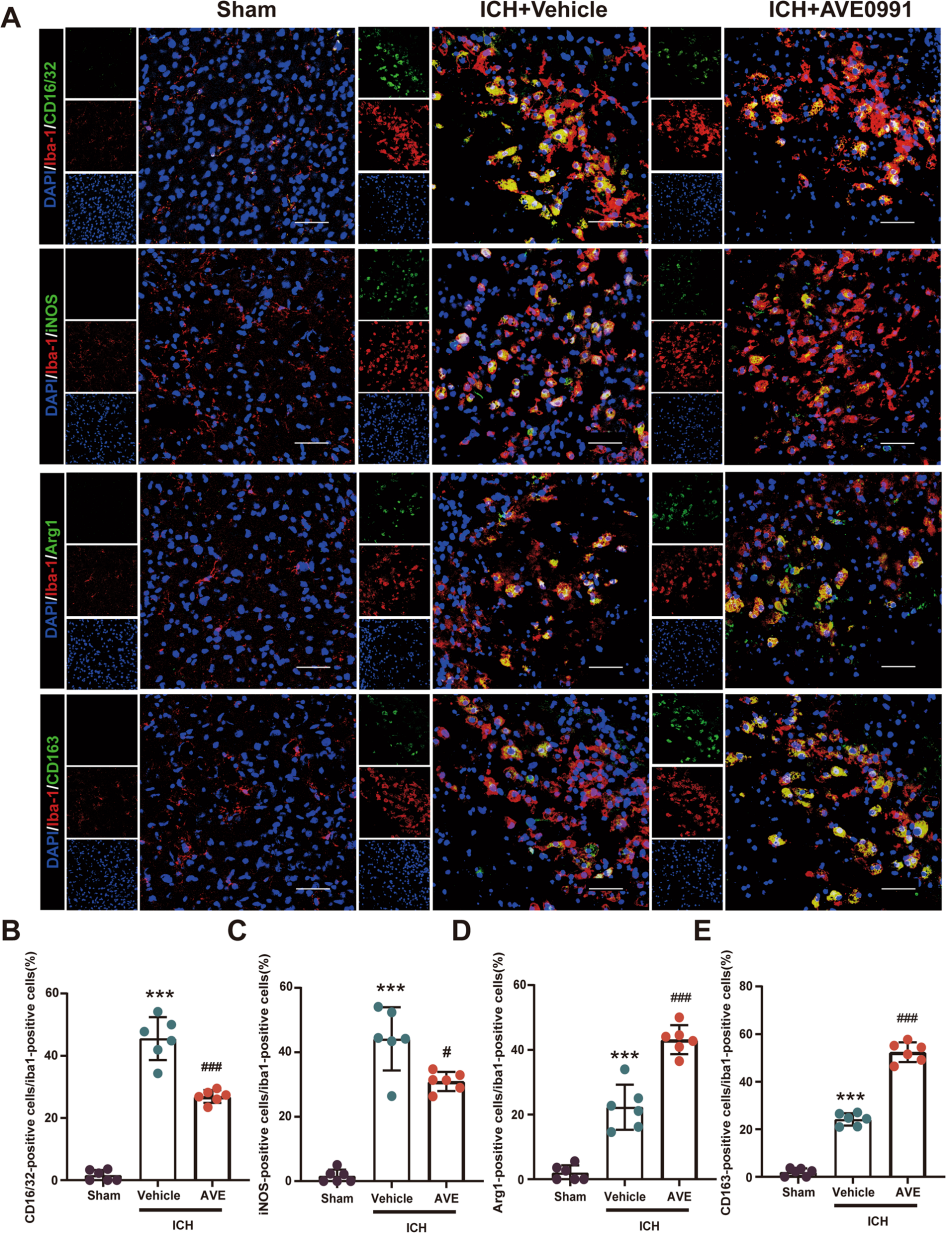
AVE0991 treatment increased the phenotypic switch of microglia/macrophages from pro‑inflammatory to regulatory phenotype. **A** Representative double immunofuorescence staining for Iba1 (green) and CD16/32 (red), iNOS (red), Arg1 (red) and CD163 (red) in the perihematomal area. Scale bar = 50 μm. **B**–**E** Quantitative analyses of Iba1 + CD16/32 microglia/macrophage, Iba1 + /iNOS + pro‑inflammatory microglia/macrophage and Iba1 + /Arg1 + , Iba1 + /CD163 + regulatory microglia/macrophage in the perihematomal area at 3 days after ICH. Data were represented as mean ± SD. ***P < 0.001 vs. Sham; ^###^P < 0.001 vs. ICH + Vehicle; n = 6/group. AVE, AVE0991

Microglia/macrophages regulate the repair process of ICH injury through functional phenotypic changes. We further investigated the functional changes of microglia/macrophages after AVE0991 administration to evaluate whether Mas receptor activation could participate in neuroprotection by regulating microglia/macrophages-mediated inflammatory response in ICH. Double immunofuorescence staining was performed and revealed AVE0991 post-treatment significantly decreased the number of CD16/32 + Iba1 + and iNOS + Iba1 + pro-inflammatory microglia/macrophages (P < 0.05, Fig. 6A–C), and significantly increased the numbers of Arg1 + Iba1 + and CD163 + Iba1 + regulatory microglia/macrophages (P < 0.05, Fig. 6A, D and E) in the perihematomal area at 3 days post-ICH. These findings demonstrated that AVE0991 post-treatment promoted the phenotypic switch of microglia/macrophages from pro-inflammatory to regulatory phenotype after ICH.

### Inhibition of Mas or Nrf2 abolished the effects of AVE0991 post‑ICH

**Fig. 7:**
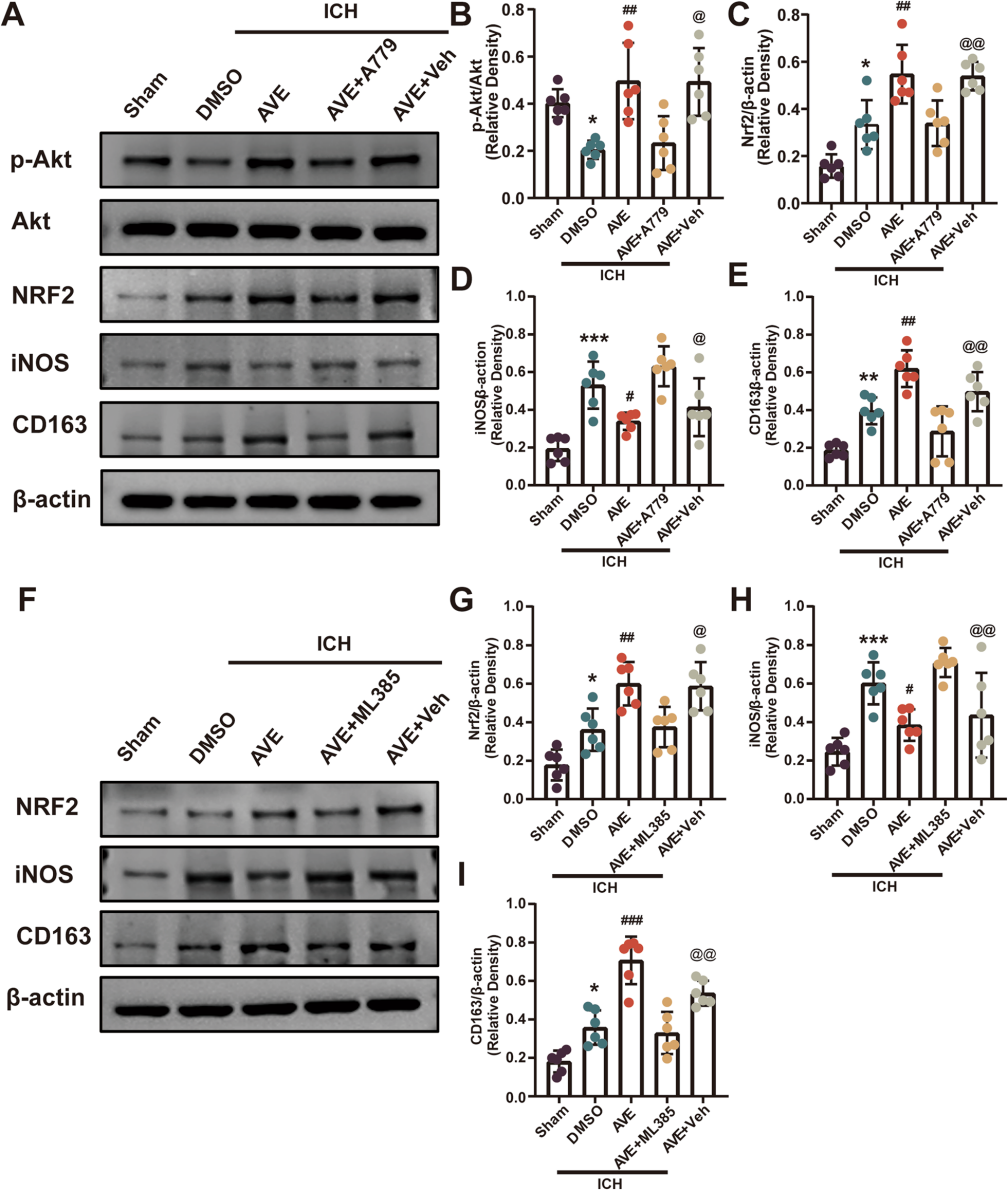
Inhibition of Mas or Nrf2 abolished the effects of AVE0991 after ICH. **A** Representative western blot bands. **B–E** quantitative analyses of p-Akt/Akt, Nrf2, iNOS, and CD163 at 3 days after ICH. *P < 0.05, **P < 0.01, ***P < 0.001 vs. Sham; ^#^P < 0.05, ^##^P < 0.01 vs. ICH + Vehicle; ^@^P < 0.05, ^@@^P < 0.05 vs. ICH + AVE + A779. **F** Representative Western blot bands. **G**–**I** Quantitative analyses of Nrf2, iNOS, and CD163 at 3 days after ICH. *P < 0.05, **P < 0.01, ***P < 0.001 vs. Sham; ^#^P < 0.05, ^##^P < 0.01, ^###^P < 0.001 vs. ICH + Vehicle; ^@^P < 0.05, ^@@^P < 0.05 vs. ICH + AVE + ML385. Data was represented as mean ± SD, n = 6/group. Veh, Vehicle; AVE, AVE0991

To understand the mechanism of the neuroprotection of Mas activation, WB analysis was performed at 3 days post-ICH to assess the Akt/Nrf2 signaling pathway and microglial markers. The expression of p-Akt, Nrf2, and CD163 increased with AVE0991 administration in ICH + AVE0991 group compared with ICH + DMSO group, while iNOS decreased (P < 0.05, Fig. 7A–E). The inhibition of Mas by A779 with AVE0991 administration significantly reversed this effect in ICH + AVE0991 + A779 group compared with ICH + AVE0991 + Vehicle group (P < 0.05, Fig. 7A–E). And Nrf2 inhibitor ML385 also reversed this treatment effect of AVE0991, showing that expression of Nrf2 and CD163 decreased and iNOS increased in ICH + AVE0991 + ML385 group compared with ICH + AVE0991 + Vehicle group (P < 0.05, Fig. 7F–I).

### The therapeutic effect of AVE0991 was dependent on the presence of microglia

To verify if the therapeutic efficacy of AVE0991 is partially contingent upon microglia, initially, we cultivated mouse HT22 hippocampal neuronal cells in vitro with conditioned medium from BV2 microglia treated with AVE0991, and neuron survival was quantified by live/dead staining. Additional file 1: Fig. S4A demonstrates the experimental design. The results exhibited that conditioned medium from AVE0991-treated microglia could notably decrease the demise of HT22 cells handled by hemin (AVE0991 vs Vehicle, P < 0.05, Additional file 1: Fig. S4B and C).

**Fig. 8:**
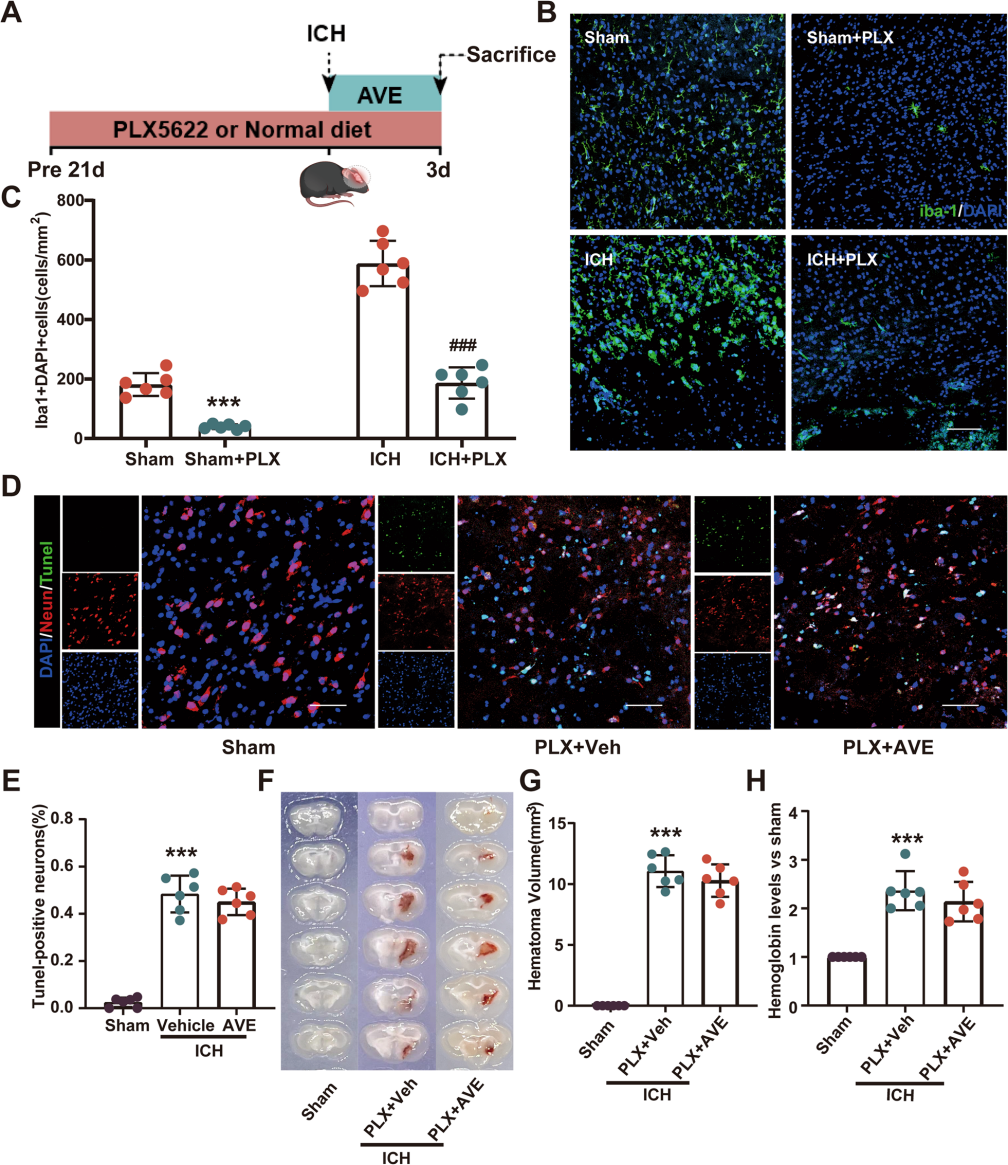
PLX5622-mediated microglia depletion attenuates the effect of AVE0991 in ICH mice. **A** Schematic diagram of PLX5622 use. **B** Representative immunofuorescence staining for Iba1 (green) in the perihematomal area after PLX5622 diet. Scale bar = 100 μm. **C** Quantification of Iba1 + microglia. ***P < 0.001 vs. Sham; ^###^P < 0.001 vs. ICH, n = 6/group. **D** Representative images of the co-localization of TUNEL (green) with neurons (NeuN, red) in the perihematomal area at 3 days after ICH. Scale bar = 50 μm. **E** Quantitative analyses of TUNEL-positive cells in the perihematomal area at 3 days after ICH (n = 6/group). **F** Representative photograph of brain sections. **G**, **H** Quantitative analyses of hematoma volume and hemoglobin level at 3 days after ICH. Data was represented as mean ± SD. ***P < 0.001 vs Sham; n = 6/group. PLX, PLX5622; Veh, Vehicle; AVE, AVE0991

Then, further in vivo experiments were conducted. The microglia in the brain of mice were eliminated using the CSF1R inhibitor PLX5622. The mice were fed a diet containing PLX5622 for 21 days to successfully deplete the microglia in the brain (Fig. 8A). Immunofluorescence staining using Iba1 demonstrated the effective elimination of Iba1 positive microglia by PLX5622 (Sham + PLX5622 vs. Sham, P < 0.05; ICH + PLX5622 vs. ICH, P < 0.05; Fig. 8B and C). To verify the protective effect of AVE0991 was mediated by microglia, we then evaluated the impact of AVE0991 on the neuroprotective effect in ICH mice following microglia depletion, the TUNEL staining results indicated that the number of neuronal apoptosis surrounding the hematoma in the ICH + PLX5622 + AVE0991 treatment group was lower than that in the ICH + PLX5622 + Vehicle group, but the difference was not significant (P > 0.05, Fig. 8D and E).

We further evaluated the impact of AVE0991 on hematoma clearance in ICH mice following microglia depletion. The results showed no significant disparity in hematoma volume between the ICH + PLX5622 + AVE0991 group and the ICH + PLX5622 + Vehicle group (P > 0.05, Fig. 8F and G). Additionally, the results of hemoglobin content were also consistent with the hematoma volume (P > 0.05, Fig. 8H), indicating that the hematoma clearance effect of AVE0991 treatment decreased significantly after microglia depletion. These findings suggested that the protective effect of AVE0991 treatment might be related to the presence of microglia.

## Discussion

ICH is considered the most perilous subtype of stroke, and effective treatments for it have not been established. The primary objectives of post-treatment include achieving clearance of hematoma and providing neuroprotection following intracerebral hemorrhage [9, 37]. Microglia/macrophages, which are the key phagocytic and neuroimmune units in the CNS, play a vital role in this process [11, 13]. Thus, promoting the microglia/macrophages' transformation towards repair phenotype represents the potential path to develop therapeutic strategies for ICH patients. In this study, as a type of G protein coupled receptor, Mas was found to be co-located with microglia/macrophages and its expression was observed to increase in activated microglia/macrophages after ICH. Activation of Mas using AVE0991 significantly enhanced the neurobehavioral outcomes, as well as facilitating promoting hematoma absorption, reducing brain edema, inhibiting peripheral neutrophil infiltration, decreasing the secretion of inflammatory factors, attenuating neuron apoptosis, and inducing a switch in microglia/macrophage phenotype from pro-inflammatory to regulatory. Our results suggested that activation of Mas might contribute to microglia’s functional phenotypic transition at least partly via Akt/Nrf2 pathway. Moreover, the elimination of microglia by PLX5622 further confirmed the protective effect of Mas activation was related to the presence of microglia.

The expression of Mas is mainly found in hippocampus, amygdala, cortex, olfactory bulb, thalamus and striatum [15, 38]. In the cellular localization of brain tissue, previous studies have found that Mas receptors could be expressed in neurons, microglia and endothelial cells [15, 38]. In the ICH model, we observed a gradual increase in Mas receptor expression in activated microglia/macrophages surrounding the hematoma through immunofluorescence staining, with peak expression at approximately 3–5 days. We further investigated the potential of the Mas receptor ligand Ang-(1–7) as a prognostic marker. In the ischemic stroke model, Lu et al. found that the expression of Ang-(1–7) in brain tissue and serum increased significantly within 48 h [39]. And in our study, significant difference in Ang-(1–7) serum expression was also observed between the 7-day post-ICH group and the Sham group.

The regulation of the Mas receptor in the treatment of nervous system diseases has been reported in literature. Mo et al. have demonstrated that activating Mas receptors could inhibit oxidative stress and neuronal apoptosis through the PKA/CREB/UCP-2 axis, thereby reducing early brain injury after subarachnoid hemorrhage [23]. The regulation of Mas axis could significantly improve neuroinflammation caused by aging through peripheral administration of AVE0991, as found by Jiang et al. [40]. In several models of middle cerebral artery occlusion in mice, Ang-(1–7) had been found to significantly reduce the infarct size and improve neurological dysfunction [39, 41–43]. However, there is currently no research available exploring the role of the AVE0991-activated Mas receptor in the setting of ICH. In this study, we administered AVE0991 intranasally to bypass the blood–brain barrier and increase entry into the brain to minimize systemic side effects associated with peripheral administration. The effective intranasal administration of AVE0991 was verified through LC–MS analysis.

For the first time, our research reported that AVE0991-activated Mas receptor promoted hematoma absorption and neuroprotection in ICH model. In present study, we observed Mas agonist AVE0991 treatment significantly reduced hematoma at 3 and 7 days after ICH. Subsequent analysis of sensorimotor function in mice post-ICH, employing a battery of neurobehavioral assessments, including mNSS, corner turning test, and FP test, unveiled striking deficits, which were closely correlated with extensive neuronal death in the perihematomal brain tissues. The profound loss of neurons and impairment of sensorimotor function in the ICH mice were remarkably reduced following treatment with AVE0991. Furthermore, we conducted a comprehensive analysis of the hippocampal CA1 region on the affected side of mice at 28 days following ICH. The remarkable effect of the AVE0991 treatment was observed through the Nissl staining, as it effectively promoted neuronal survival in the CA1 region. This finding significantly contributed to the improved long-term behavioral performance observed in mice administered with AVE0991 following ICH.

Microglia are endogenous immune cells in the CNS that, along with monocyte-derived macrophages, constitute the most important phagocytic system, playing a crucial role in neuroinflammation. Following ICH, microglia/macrophages rapidly mobilize and converge at the site of injury, secreting various cytokines to mediate the inflammatory response and expressing phagocytic receptors, performing their phagocytic function. The phenotype of microglia/macrophages is highly adaptable and they play diverse roles by regulating functional phenotypes throughout the different stages of injury [11–13]. Thus, modulating the functional phenotype of microglia/macrophages to enhance hematoma clearance, alleviate neuroinflammation and inhibit neuron apoptosis proves to be a promising approach in the treatment in brain injury. Here, we confirmed that AVE0991 administration activating Mas significantly reduced pro-inflammatory microglia/macrophages and increased regulatory microglia/macrophages at 3 days after ICH. The latest research challenged the inflexible categorization of microglia/macrophages [44]. Given their varying roles in distinct phases of brain injury, gaining a deeper understanding of the characteristics and dynamic changes of microglia/macrophages during different stages will aid in more precise regulation and treatment of brain injury. Recent advances in molecular biology and genetics have revealed the complexity of microglial phenotypes, demonstrating that these cells operate across a spectrum of activation states rather than conforming to the strict M1 or M2 categories [44, 45]. While it can be convenient to refer to “good” or “bad” microglial phenotypes in a broad sense, it is crucial to acknowledge the oversimplification of this approach. The dynamic and context-dependent nature of microglial activation underscores the importance of moving beyond the M1/M2 paradigm to better understand the nuanced roles of microglia in the CNS. Although our experiments have demonstrated the beneficial effects of AVE0991 therapy in regulating microglia/macrophages at 3 days after ICH, the optimal timing for drug administration requires evaluation, and further investigation of the dynamic features of microglial functional changes is necessary to maximize the benefits of regulating microglial phenotype.

Moreover, we further investigated the potential mechanism underlying AVE0991-triggered Mas activation, which leaded to hematoma absorption and inhibition of neuroinflammation following ICH. Previous studies have demonstrated that Ang-(1–7) activated Mas via the Akt-dependent pathway, resulting in the stimulation of eNOS activation, which triggered the production of NO and increased the level of cGMP [21]. Xu et al. observed that Ang-(1–7) upregulated p-eNOS/eNOS expression while downregulated iNOS expression through the Mas/Akt signaling pathway, which subsequently inhibited oxidative stress [46]. Previous studies have found that phosphorylation of Akt had the ability to reduce cellular apoptosis and ease neuroinflammation in diverse pathophysiological conditions [47–49]. On the other hand, Akt signaling has been reported to regulate the Nrf2 transcription factors, which aid in suppressing oxidative stress and negatively modulating the inflammatory response [27–29, 50]. Numerous studies have indicated that Nrf2 contributed to modulating the functional phenotype of microglia and enhancing microglial phagocytosis in brain injury [26, 51–54]. In present study, our data demonstrated that the administration of AVE0991 activating Mas receptor increased phosphorylation of Akt and expression of Nrf2 signaling pathways. These findings showed that AVE0991 treatment induced Mas receptor activation, leading to enhanced hematoma absorption and neuroprotection, in part by modulating the Akt/Nrf2 signaling cascade. To investigate the dependency of AVE0991’s protective effect on microglia presence following ICH, we conducted brain-wide elimination of microglial cells using PLX5622 [55]. Our results showed that the protective effect of AVE0991 was diminished in the absence of microglia. These findings reinforced our conclusion that the activation of the Mas receptor by AVE0991 played a crucial role in hematoma elimination and neuroprotection following ICH by regulating the transformation of microglia into anti-inflammatory, phagocytic, and reparative phenotypes.

Inhibiting neuroinflammation and promoting hematoma clearance are crucial aspects of treating ICH, and microglia appear to be most intimately involved in ICH-induced neuronal damage. While we have validated the role of Mas receptors in regulating anti-inflammatory and phagocytic functions in microglia, their role in neurons should not be overlooked. Numerous studies have indicated the significance of Mas receptors in neuronal cells [23, 42, 56, 57]. Previous study has also reported that during neuronal injury, although the activation of the neuronal Ang-(1–7)/Mas axis provided a certain compensatory protective effect, additional neurotoxins and the activation of microglial cells may overwhelm the neuronal compensatory mechanism [58]. Therefore, in severe pathological processes such as ICH with intense neuroinflammation and hematoma formation, regulating microglia may exhibit a more pronounced effect. This partially explains the significant reduction in the protective effect of Mas after removing microglia in our study. However, even after the deletion of microglia, our TUNEL experiments with AVE0991 treatment still showed a trend of neuroprotection, albeit without reaching statistical significance. Future research still needs to further elucidate more mechanisms by which Mas receptors regulate neuroinflammation and neuroprotection, providing a more solid theoretical basis for the development of clinical drugs.

There are some limitations should be acknowledged in this study. First, based on the existing research, Mas receptors are primarily expressed in microglia, neurons, and vascular endothelial cells within the CNS. And we have also observed the expression of Mas in astrocytes surrounding the brain hematoma. Therefore, it is necessary to further investigate the expression of Mas receptors in vascular endothelial cells, astrocytes and neurons, and explore their potential role in the pathological mechanisms in the setting of ICH. Second, we focused on the role of Mas in hematoma absorption and neuroinflammation, further exploration of other protective roles of Mas, such as blood–brain barrier protection, after ICH is needed. Third, we have validated a mechanism of the Akt/Nrf2 signaling pathway in the Mas-mediated protective effect following ICH. However, future studies are required to elucidate other signaling pathways involved in the anti-neuroinflammatory process. Finally, it should be noted that this study only used male mice and did not address potential gender differences, which is another limitation that should be pointed out.

## Conclusion

**Fig. 9:**
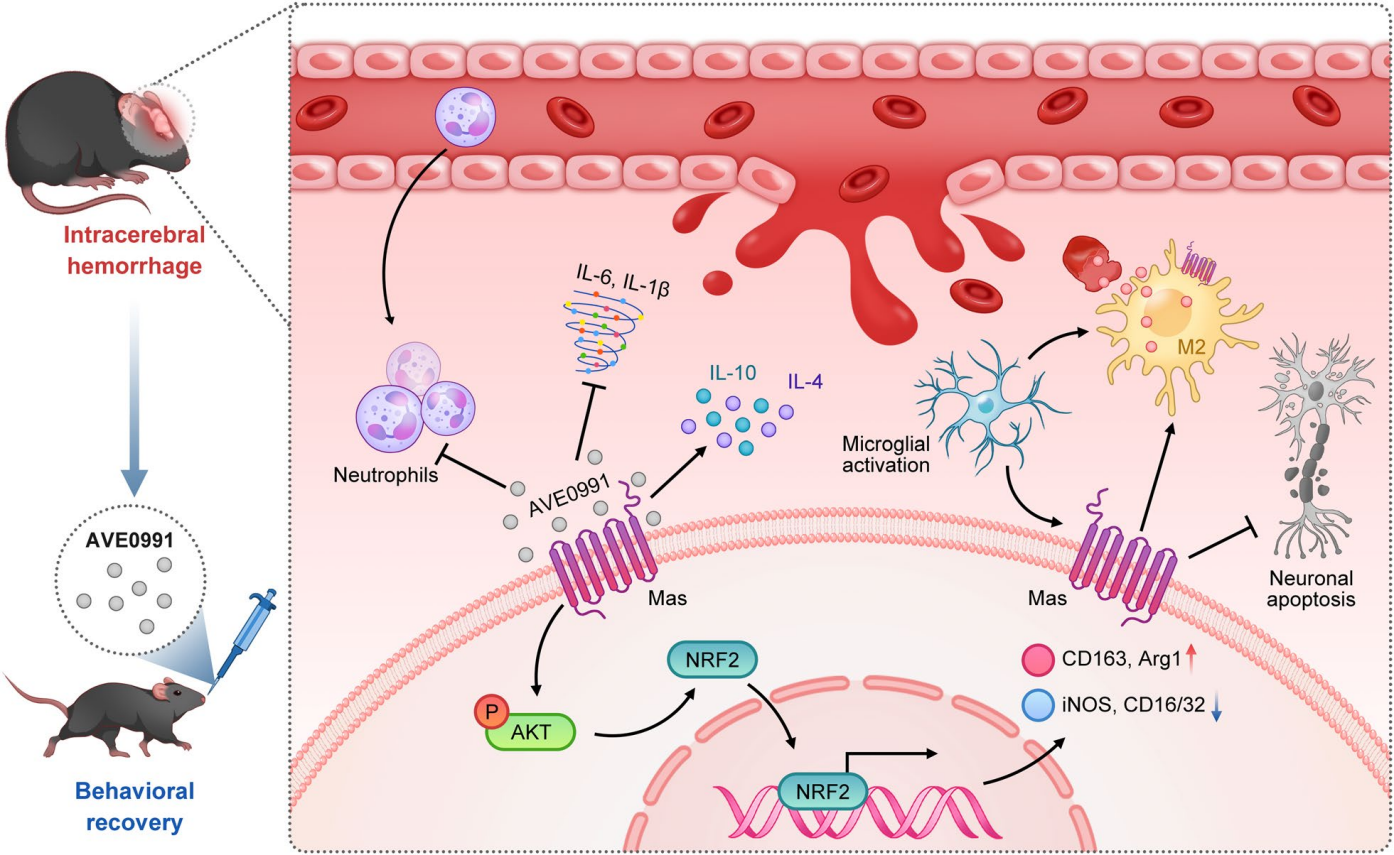
A schematic diagram showing the hematoma clearance and neuroprotective effect of Mas activation by AVE0091

In summary, we first demonstrated that the administration of AVE0991 activating Mas receptor improved neurological deficits, promoted hematoma absorption, reduced brain swelling, alleviated neuroinflammation, and neuronal apoptosis, partially through the Akt/Nrf2 signaling pathway following ICH (Fig. 9). Therefore, Mas activation using AVE0991 may provide a promising therapeutic strategy in clinical management of ICH patients.

### Supplementary Information

**Additional file 1: Figure S1.** Experimental design and animal groups. ICH, intracerebral hemorrhage; ELISA, enzyme-linked immunosorbent assay; IF, immunofuorescence staining; LC–MS, Liquid Chromatograph-Mass Spectrometer; WB, western blot; TUNEL, transferase dUTP nick end labeling; FJC, Fluoro-Jade C staining; qPCR, quantitative real-time polymerase chain reaction. **Figure S2.** LC–MS results of standard AVE0991 and brain tissue. **A** Chromatogram of standard AVE0991; **B** Ion fragmentation map of standard AVE0991; **C** Chromatogram of brain tissue; **D** Ion fragmentation map of brain tissue; **E**, **F** Chemical structure of decomposition products of AVE0991. **Figure S3.** Determine the optimal dose of AVE0991 for ICH treatment. Quantitative analyses of mNSS score at 7 days after ICH. ***P < 0.001 vs Sham; ^##^P < 0.01 vs Vehicle; n = 6/group. **Figure S4.** AVE0991 reduces neuronal death after exposure to hemin in a microglia-dependent manner. **A** Experimental design; (**B**) survival (green) and death (red) staining of cultured neurons; and **C** quantitative analysis of the percentage of dead neurons. ***P < 0.001 vs. Control; ^#^P < 0.05 vs. Vehicle, scale = 50 μm.

## Data Availability

The data, analytic methods, and study materials will be made available to other researchers to reproduce the results or replicate the procedures. The data that support the findings of this study are available from the corresponding author upon reasonable request. Authors will be responsible for maintaining availability.

## Abbreviations

ICH: Intracerebral hemorrhage
RAS: Renin-angiotensin system
ACE: Angiotensin converting enzyme
PLA2: Phospholipase A2
PI3K/Akt: Phosphatidylinositol 3-kinase/protein kinase B
Nrf2: Nuclear factor E2-related factor 2
mNSS: Modified neurological deficit score
MPO: Myeloperoxidase
IL-1β: Interleukin-1β
i.n.: Intranasal
i.p.: Intraperitoneal
LC–MS: Liquid Chromatograph-Mass Spectrometer
DMSO: Dimethylsulfoxide
GFAP: Glial fbrillary acidic protein
Iba-1: Ionized calcium-binding adaptor molecule-1
NeuN: Neuronal nuclear
PBS: Phosphate-bufered saline
i.c.v.: Intracerebroventricular
FJC: Fluoro-Jade C
TUNEL: Terminal deoxynucleotidyl transferase dUTP nick end labeling
CNS: Central nervous system
ELISA: Enzyme‑linked Immunosorbent Assay
q-PCR: Quantitative Real-time Polymerase Chain Reaction
