# Supplementary material for: Mas receptor activation facilitates innate hematoma resolution and neurological recovery after hemorrhagic stroke in mice
Source: J Neuroinflammation. 2024 Apr 24;21:106. doi: 10.1186/s12974-024-03105-8 (PMC11041011; doi:10.1186/s12974-024-03105-8)
Supplement: Supplementary file 1 — Additional file 1: Figure S1. Experimental design and animal groups. ICH, intracerebral hemorrhage; ELISA, enzyme-linked immunosorbent assay; IF, immunofuorescence staining; LC–MS, Liquid Chromatograph-Mass Spectrometer; WB, western blot; TUNEL, transferase dUTP nick end labeling; FJC, Fluoro-Jade C staining; qPCR, quantitative real-time polymerase chain reaction. Figure S2. LC–MS results of standard AVE0991 and brain tissue. A Chromatogram of standard AVE0991; B Ion fragmentation map of standard AVE0991; C Chromatogram of brain tissue; D Ion fragmentation map of brain tissue; E, F Chemical structure of decomposition products of AVE0991. Figure S3. Determine the optimal dose of AVE0991 for ICH treatment. Quantitative analyses of mNSS score at 7 days after ICH. ***P < 0.001 vs Sham; ##P < 0.01 vs Vehicle; n = 6/group. Figure S4. AVE0991 reduces neuronal death after exposure to hemin in a microglia-dependent manner. A Experimental design; (B) survival (green) and death (red) staining of cultured neurons; and C quantitative analysis of the percentage of dead neurons. ***P < 0.001 vs. Control; #P < 0.05 vs. Vehicle, scale = 50 μm. [file 12974_2024_3105_MOESM1_ESM.docx]

**Supplementary materials**

**Supplementary Part I**

In the present study, all mice were randomly assigned to the following experiments.


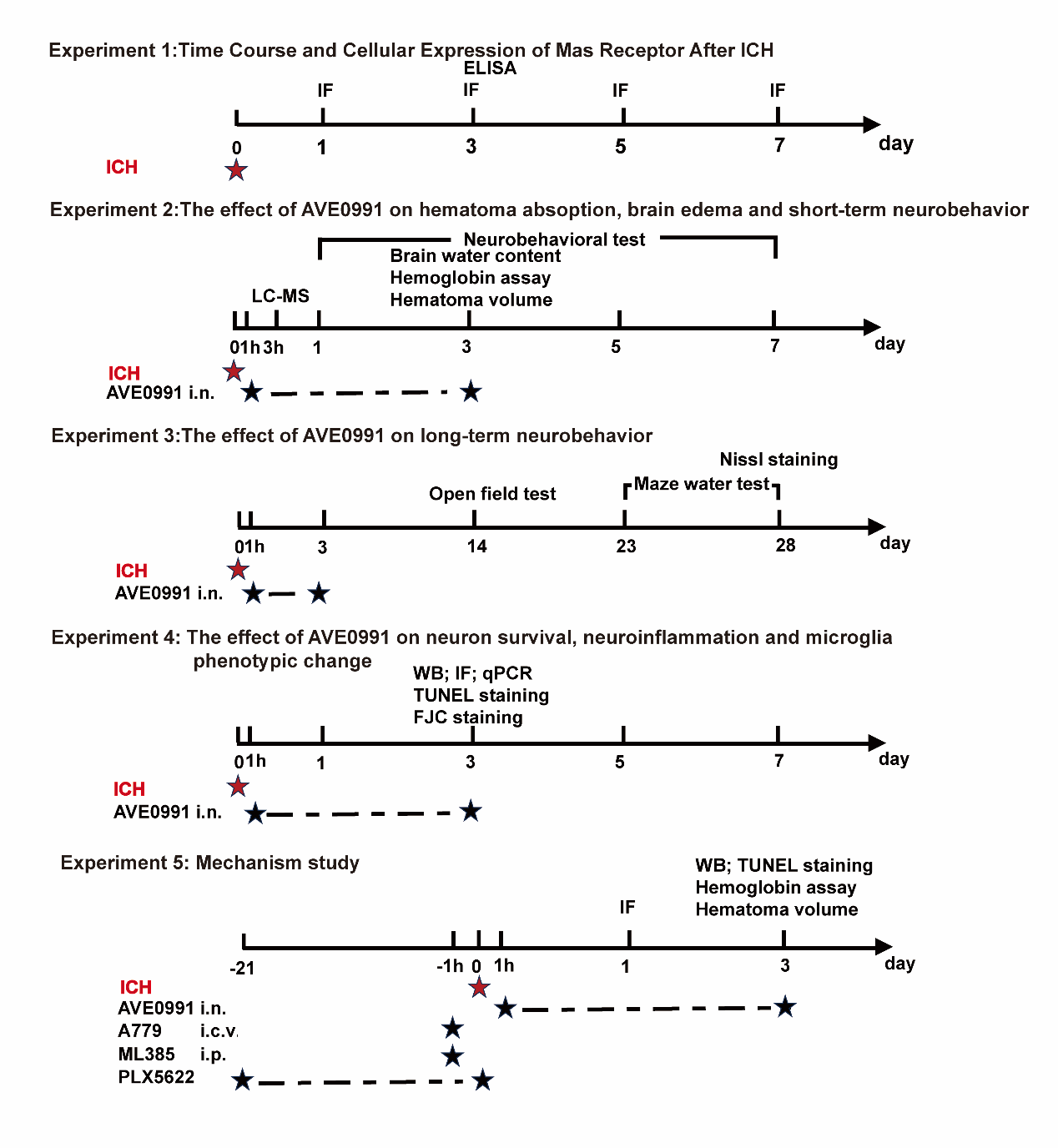


Figure S1. Experimental design and animal groups. ICH, intracerebral hemorrhage; ELISA, enzyme-linked immunosorbent assay; IF, immunofuorescence staining; LC-MS, Liquid Chromatograph-Mass Spectrometer; WB, western blot; TUNEL, transferase dUTP nick end labeling; FJC, Fluoro-Jade C staining; qPCR, quantitative real-time polymerase chain reaction.

**Experiment 1**

Given that the primary cells responsible for hematoma clearance are microglia/macrophages, we performed to assess Mas expression in microglia/macrophages, and 25 mice were randomly assigned to 5 groups: Sham, 1, 3, 5 and 7 days after ICH for immunofluorescence staining. Enzyme-linked Immunosorbent Assay (ELISA) was conducted to evaluate the concentration of Ang-(1-7) in the brain tissue around hematoma in the sham group and the 3-day group (n = 4/group). Additional 18 mice (n=8 in Sham and n=10 in 3-day ICH group) were used for detecting its serum levels. The cellular localization of Mas was assessed using double-labeling immunofluorescence staining to co-localize Mas with ionized calcium binding adapter molecule 1 (iba-1), glial fibrillary acidic protein (GFAP), neuronal specific nuclear protein (NeuN) and CD31 at 3 days after ICH.

**Experiment 2**

2 mice were used for liquid chromatograph-mass spectrometer (LC-MS) analysis to confirm whether AVE0991 entered the brain tissue following intranasal administration. 30 mice were used to evaluate the efficacy of three different doses of AVE0991(0.3mg/kg, 0.9 mg/kg, 2.7mg/kg) to determine the optimal dose. The mNSS was evaluated at 7 days after ICH.18 mice were randomly assigned to three groups (n=6/group): Sham; ICH+Vehicle (10% DMSO), ICH+AVE0991 (0.9 mg/kg), short-term neurological tests were measured at 1, 3, 5, and 7 days after ICH. For effects of AVE0991 on hematoma absorption after ICH, 30 mice were randomly assigned into five groups: Sham, ICH+Vehicle (10% DMSO, 3 days), ICH+AVE0991 (0.9 mg/kg, 3 days), ICH+Vehicle (10% DMSO, 7 day), and ICH+AVE0991 (0.9 mg/kg, 7 day), hematoma volume and hemoglobin content were evaluated. For brain edema assessment, brain water content was determined at 3 days after ICH in three groups: Sham, ICH+Vehicle (10% DMSO), ICH+AVE0991 (0.9 mg/kg).

**Experiment 3**

To assess the long-term neurobehavioral outcomes after ICH, a total of 18 mice were randomly divided into three groups (n=6/group): Sham, ICH+Vehicle (10% DMSO), and ICH+AVE0991 (0.9 mg/kg). On 14 days after ICH, the open field test was conducted to assess autonomic activity and exploratory behavior of mice. On 23-28 days post-ICH, the Morris water maze test was performed to evaluate spatial cognitive functions.

**Experiment 4**

To investigate effects of AVE0991 on neuronal apoptosis, neutrophil infiltration, inflammatory cytokines release and microglia/macrophage functional phenotypic transition at 3 days after ICH, a total of 54 mice were randomized into three groups (n=6/group): Sham, ICH+Vehicle (10% DMSO) and ICH+AVE0991 (0.9 mg/kg). Among these mice, 18 mice were randomly divided into three groups (n=6/group) for immunofluorescence staining of IL-1β, and myeloperoxidase (MPO), Fluoro-Jade C staining, and terminal deoxynucleotidyl transferase dUTP nick end labeling (TUNEL) co-staining with neuronal marker (NeuN). Microglia/macrophage functional phenotypic transition were evaluated by double immunofluorescence staining. To quantify the protein levels of Iba-1, IL-1β, MPO, Bax and Bcl-2, an additional 18 mice were randomly divided into three groups for western blot analysis (n=6/group): Sham, ICH+Vehicle (10% DMSO), ICH+AVE0991 (0.9 mg/kg). Further, another 18 mice were randomly divided into three groups for qPCR analysis (n=6/group) for IL-1β, IL-6, IL-4 and IL-10.

**Experiment 5**

Mechanism study (Mas/Akt/Nrf2 signaling pathway). To investigate the potential molecular mechanism of Mas activation, 30 mice were randomly divided to five groups (n=6/group): Sham, ICH+Vehicle (10% DMSO), ICH+AVE0991 (0.9 mg/kg), ICH+AVE0991 (0.9 mg/kg)+A779 (30μmol/ml), ICH+AVE0991 (0.9 mg/kg)+ Vehicle (PBS). To further explore the Nrf2 signaling pathway in Mas-mediated microglia/macrophage functional phenotypic transition after ICH, another 30 mice were randomized into five groups (n=6/group): Sham, ICH+Vehicle (10% DMSO), ICH+AVE0991 (0.9 mg/kg), ICH+AVE0991 (0.9 mg/kg)+ML385(30 mg/kg), ICH+AVE0991 (0.9 mg/kg)+Vehicle (5% DMSO). Western blots were performed at 3 days after ICH induction.

**Experiment 6**

To verify the protective effect of AVE0991 was mediated by microglia, we cultivated mouse HT22 hippocampal neuronal cells in vitro with conditioned medium from BV2 microglia treated with AVE0991, and neuron survival was quantified by dead/live staining. Then, further in vivo experiments were conducted. The microglia in the brain of mice were eliminated using the CSF1R inhibitor PLX5622. To determine the PLX5622 efficiency, 24 mice (n=6/group) were randomly allocated into 4 groups: Sham, Sham+PLX5622, ICH and ICH+PLX5622. Then, the impact of AVE0991 on hematoma clearance and neuron survival in ICH mice following microglia depletion were evaluated. 18 mice (n=6/group) were randomly allocated into 3 groups including Sham, ICH+AVE0991 (0.9mg/kg)+PLX5622, and ICH+Vehicle (10% DMSO)+PLX5622 for TUNEL co-staining with NeuN. And another 18 mice were evaluated hematoma volume and hemoglobin content.

**Supplementary Part II**

Primers for qPCR

Primers (5’-3’)

IL-6 Forward primer TTCTTGGGACTGATGCTGGTG

IL-6 Reverse primer GCCATTGCACAACTCTTTTCTC

IL-1β Forward primer GCATCCAGCTTCAAATCTCGC

IL-1β Reverse primer TGTTCATCTCGGAGCCTGTAGTG

IL-10 Forward primer AATAAGCTCCAAGACCAAGGTGT

IL-10 Reverse primer CATCATGTATGCTTCTATGCAGTTG

IL-4 Forward primer GATAAGCTGCACCATGAATGAGT

IL-4 Reverse primer CCATTTGCATGATGCTCTTTAGG

GAPDH Forward primer CCTCGTCCCGTAGACAAAATG

GAPDH Reverse primer TGAGGTCAATGAAGGGGTCGT

**Supplementary Part III**


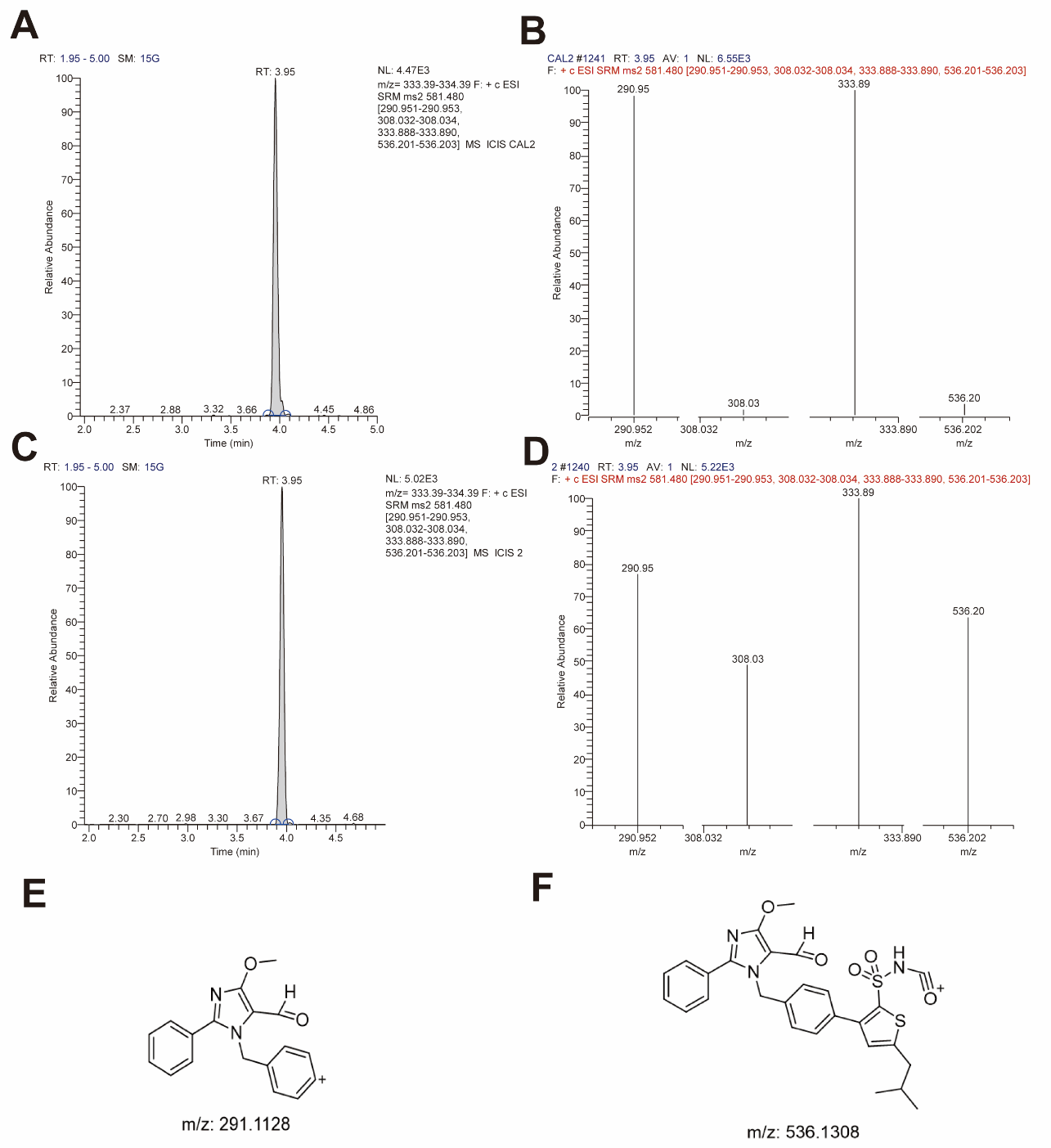


Figure S2 LC-MS results of standard AVE0991 and brain tissue. A Chromatogram of standard AVE0991; B Ion fragmentation map of standard AVE0991; C Chromatogram of brain tissue; D Ion fragmentation map of brain tissue; E-F Chemical structure of decomposition products of AVE0991.


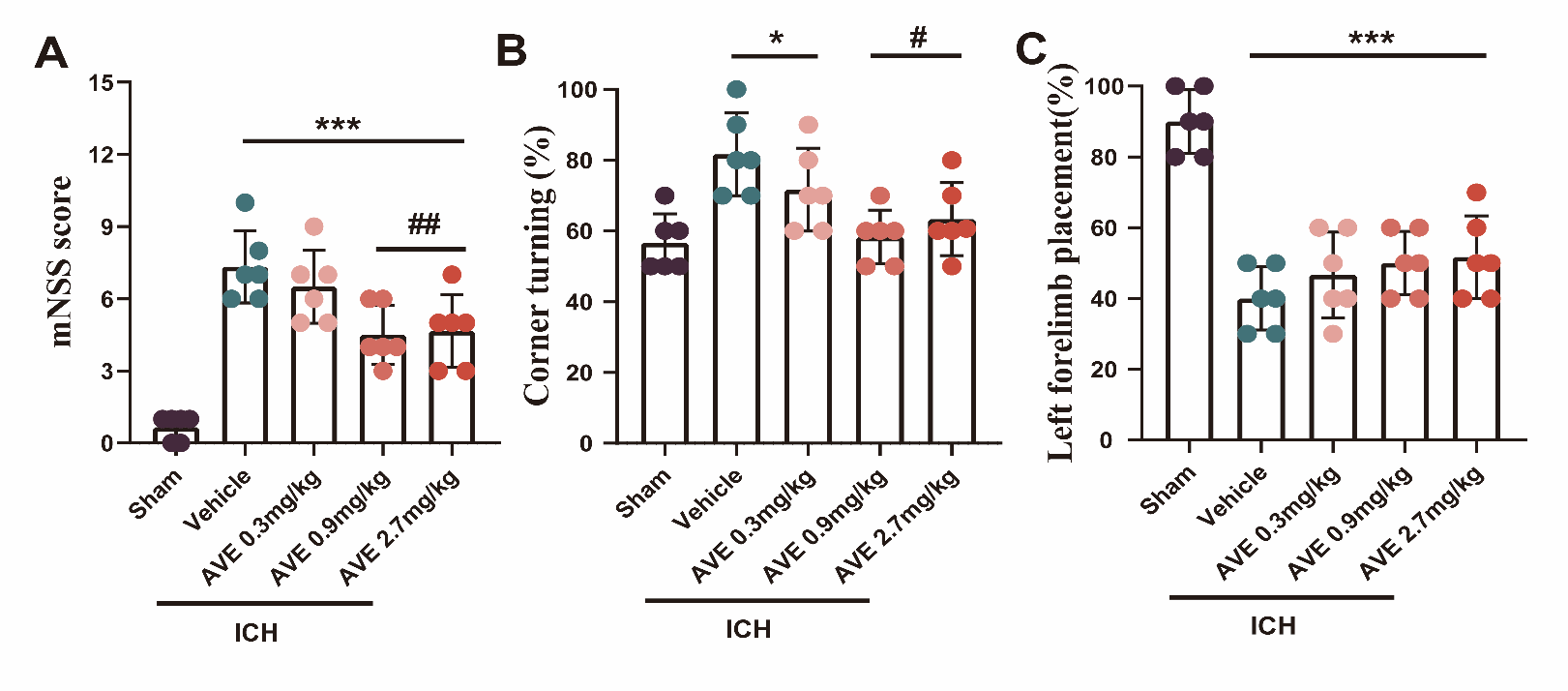


Figure S3 Determine the optimal dose of AVE0991 for ICH treatment. Quantitative analyses of mNSS score at 7 days after ICH. ***P<0.001 vs Sham; ##P<0.01 vs Vehicle; n=6/group.


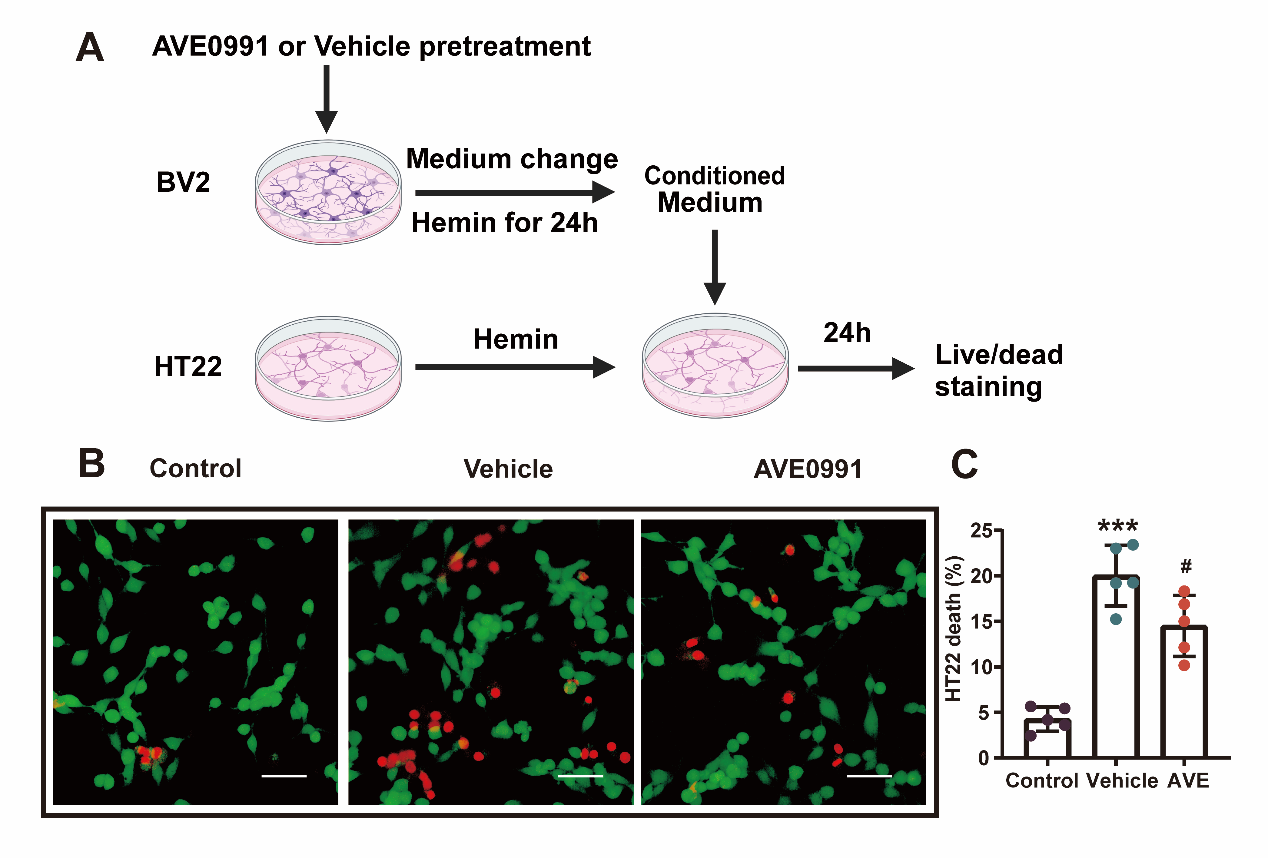


Figure 4S Figure 4-4 AVE0991 reduces neuronal death after exposure to hemin in a microglia-dependent manner. (A) experimental design; (B) survival (green) and death (red) staining of cultured neurons; and (C) quantitative analysis of the percentage of dead neurons. ***P < 0.001 vs. Control; #P <0.05 vs. Vehicle, scale=50μm.
